# Smorgasbord or symphony? Assessing public health nutrition policies across 30 European countries using a novel framework

**DOI:** 10.1186/1471-2458-14-1195

**Published:** 2014-11-21

**Authors:** Ffion Lloyd-Williams, Helen Bromley, Lois Orton, Corinna Hawkes, David Taylor-Robinson, Martin O’Flaherty, Rory McGill, Elspeth Anwar, Lirije Hyseni, May Moonan, Mike Rayner, Simon Capewell

**Affiliations:** Department of Public Health & Policy, Institute of Psychology, Health & SocietyUniversity of Liverpool, Whelan Building, Quadrangle, L69 3GB Liverpool, Merseyside, UK; World Cancer Research Fund International, London, UK; University of Oxford, Oxford, UK

**Keywords:** Public health nutrition, Public health policy, Europe, Food policy mapping, Qualitative

## Abstract

**Background:**

Countries across Europe have introduced a wide variety of policies to improve nutrition. However, the sheer diversity of interventions represents a potentially bewildering smorgasbord.

We aimed to map existing public health nutrition policies, and examine their perceived effectiveness, in order to inform future evidence-based diet strategies.

**Methods:**

We created a public health nutrition policy database for 30 European countries . National nutrition policies were classified and assigned using the marketing "4Ps" approach *Product* (reformulation, elimination, new healthier products); *Price* (taxes, subsidies); *Promotion* (advertising, food labelling, health education) and *Place* (schools, workplaces, etc.).

We interviewed 71 senior policy-makers, public health nutrition policy experts and academics from 14 of the 30 countries, eliciting their views on diverse current and possible nutrition strategies.

**Results:**

*Product* Voluntary reformulation of foods is widespread but has variable and often modest impact. Twelve countries regulate maximum salt content in specific foods.

Denmark, Austria, Iceland and Switzerland have effective trans fats bans.

*Price* EU School Fruit Scheme subsidies are almost universal, but with variable implementation.

Taxes are uncommon. However, Finland, France, Hungary and Latvia have implemented ‘sugar taxes’ on sugary foods and sugar-sweetened beverages. Finland, Hungary and Portugal also tax salty products.

*Promotion* Dialogue, recommendations, nutrition guidelines, labelling, information and education campaigns are widespread. Restrictions on marketing to children are widespread but mostly voluntary.

*Place* Interventions reducing the availability of unhealthy foods were most commonly found in schools and workplace canteens.

Interviewees generally considered mandatory reformulation more effective than voluntary, and regulation and fiscal interventions much more effective than information strategies, but also politically more challenging.

**Conclusions:**

Public health nutrition policies in Europe appear diverse, dynamic, complex and bewildering. The "4Ps" framework potentially offers a structured and comprehensive categorisation.

Encouragingly, the majority of European countries are engaged in activities intended to increase consumption of healthy food and decrease the intake of "junk" food and sugary drinks. Leading countries include Finland, Norway, Iceland, Denmark, Hungary, Portugal and perhaps the UK. However, all countries fall short of optimal activities. More needs to be done across Europe to implement the most potentially powerful fiscal and regulatory nutrition policies.

**Electronic supplementary material:**

The online version of this article (doi:10.1186/1471-2458-14-1195) contains supplementary material, which is available to authorized users.

## Background

Non-communicable diseases account for over 85% of deaths in Europe [1], and poor diet is responsible for up to 40% of the non-communicable disease (NCD) burden [2]. Achieving optimal diet strategies could halve the cardiovascular disease burden, and substantially reduce other NCDs [3, 4]. Food policies are thus under increasing scrutiny in Europe.

Previous studies reviewing European public health nutrition policies are informative yet limited to specific actions and public health nutrition topics. The EuroHeart I study identified cardiovascular prevention strategies in 16 European countries [5] spanning tobacco, public health, physical activity, and food, including some legislative and policy action. The Eatwell project reviewed and evaluated European national healthy eating policies, but focussed mainly on public information campaigns, regulation of meals at schools/canteens and nutrition education programmes. The authors also highlighted the need to measure and compare effectiveness between countries [6].

The PORGROW study examined policy options for responding to the growing challenge from obesity. Respondents from nine EU states indicated that various interventions are required. Interestingly, the costs of various policy options were deemed less important than their social and health benefits, efficacy, acceptability and practical feasibility [7].

The WHO 8 Country study uncovered a wealth of material to support continued development of the implementation of the European NCD Strategy as a flexible policy framework. It thus provided a valuable learning experience for the further policy analyses [8].

The WHO Global nutrition policy review analysed information from 119 WHO Member States including the European region, on the presence of nutrition policies and programmes, topics covered, implementation, the key stakeholders the existence of coordination mechanisms, and monitoring and evaluation processes [9]. The review found most countries had policies and programmes that are addressing key nutrition issues, such as obesity and diet-related NCDs. However, gaps were identified in the design, content and implementation of nutrition policies and programmes [9].

The WHO NOPA database project subsequently aimed to monitor progress on nutrition, obesity and physical activity. At present, the database covers all 53 Member States in the WHO European Region, providing limited information on policy documents, budgets, and any coordinating mechanisms [10]. The Public Health Evaluation and Impact Assessment Consortium noted that much additional data exists in the database, but is not yet publically available [11].

The recent evaluation of the Strategy for Europe on Nutrition, Overweight and Obesity [11] concluded that progress in the development and implementation of nutrition policies has been ‘reasonably effective’ – within and between countries; however, it has been uneven. Furthermore, most of the action taken thus far at EU and national levels has been ‘soft’: providing information; limited interventions in schools; and voluntary actions by the food industry. Worryingly, the report cautions that without a new stimulus, political interest at the European level will fade [11].

These studies therefore all highlight that although a variety of policies intended to improve nutrition have been introduced across Europe, their potential effects are not easy to assess. Furthermore, the diverse range of diet interventions represents a potentially bewildering "policy cacophony", a smorgasbord which is difficult to comprehend, categorise or evaluate. As part of the wider EuroHeart II project, we therefore aimed to identify and map public health nutrition policies across Europe, categorise them using a novel "4Ps" framework, and assess their perceived effectiveness. Our findings might then contribute to the debate concerning future evidence-based dietary strategies to prevent cardiovascular disease and other non-communicable diseases.

## Methods

We used a mixed methods comparative study design. A quantitative approach was used to map public health nutrition policy actions in all 30 Western and Central European countries. Semi-structured interviews were then conducted to provide rich detail around the extent of implementation of specific policies, and progress towards national targets.

### Study protocol

A study protocol (Additional file 1) was written as part of the larger EuroHeart II study [12]. The protocol outlined: 1. Specific objectives. 2. Definition of Policies and policy documents (policies relating to CVD and other NCD prevention in relation to food; written documents that contain strategies and priorities, with defined goals and objectives and are issued by a public administration). 3. Approaches for collecting policy documents. 4. Inclusion and exclusion criteria. 5. Analysis and reporting.

### Quality control measures

An advisory group was established comprising the project team together with experts in quantitative and qualitative methodology, and food policy and public health policy analysis. Meeting monthly, quality control was sustained by group discussion upon the design and development of data collection tools, project progress, analysis and reporting of findings. The advisory group ensured that the project timetable was maintained, any issues or problems regarding recruitment and emerging findings were discussed and resolved and appropriate dissemination of findings. Furthermore, interview transcripts were analysed by at least two project researchers independently and discrepancies in interpretation of data was discussed until consensus was reached.

### Conceptual framework

To ensure a coherent approach to the mapping of policies in 30 European countries, we developed a conceptual framework. After extensive piloting and reviewing, we agreed the most practical and coherent approach was the "4Ps" marketing mix framework: Product, Price, Promotion and Place (Table 1). An approach used by producers and marketers to systematically assess how well products match their target markets [13, 14].

**Table 1:** **The "4Ps" marketing mix applied to public health nutrition policies**

**Price**	Taxes; subsidies; or other economic incentives
**Product**	Reformulation; elimination or new products
**Place**	Schools, workplaces or community settings
**Promotion**	Restricting marketing to children and adults (advertising controls); nutritional food labelling; nutritional information on menus; public information campaigns; and health education

("Multi-component interventions" might involve a combination of several approaches).

### Mapping exercise

We identified, extracted and categorised public health nutrition policy actions in 30 Western and Central European countries (All EU 27 countries, plus Switzerland, Norway and Iceland) and summarised them in an Excel database. We sought to identify key policy documents in the 30 European countries by searching policy documents, grey literature, nationally important websites (e.g. National Institutes of Public Health, Ministries of Health), and National Nutrition Councils and the WHO European Nutrition, Obesity and Physical Activity (NOPA) database.

The *inclusion criteria* were government endorsed policy documents covering cardiovascular disease prevention policies or chronic disease in relation to food (e.g. food labelling, legislation on food fat, sugar and salt content etc.) and health focussed taxation or subsidies. Policy documents included: National Acts, Laws, Legislation, Ministerial Decrees (or equivalent); National policies/strategies or plans; and policies/strategies or plans in preparation; social, economic and agricultural policies with a direct effect upon public health nutrition; documents available in English. Information was also collected on the EU school fruit and school milk schemes.

The *exclusion criteria* were policies relating to micronutrients; policies developed and/or implemented at local/regional level; polices that had not been implemented.

National policy actions were classified according to the "4Ps" conceptual framework .

### Interviews with key informants

To validate the policy database, we conducted interviews with 71 national experts from 14 countries (Belgium, Czech Republic, England, Estonia, Finland, Germany, Greece, Iceland, Italy, Ireland, Malta, Poland, Portugal and Slovenia). The 14 diverse countries were selected as a geographical representation of the 30 European countries, i.e. North, South, East and West. (Limited time and resources prevented us from conducting interviews across all 30 countries). The interviews elicited informant’s views on a wide range of potential national public health nutrition polices, initially with a focus on cardiovascular prevention. Interview questions were developed and piloted with key experts in England.

We identified senior food policy makers and topic experts in each country by purposive sampling.

We used various sources including the European Heart Network, national Heart Foundations, the published literature, key websites and ‘snowballing’ via expert colleagues and networks. We invited potential participants by email, explaining the project and requesting their participation as a national expert in public health nutrition policy. Prior to interview, participants received an information sheet, a consent form, the interview questions and a written summary of public health nutrition policies and related initiatives in their country. The latter two enabled familiarity with the interview content and format. We conducted the subsequent interviews in English, in person, by telephone or Skype. All interviews were digitally recorded and typically lasted from 45 to 60 minutes.

The interviews were transcribed and entered into NVIVO software. A set of broad codes were initially created based upon the interview guide and research objectives. Transcripts were then coded line by line using an inductive method of open coding; whereby researchers allowed patterns and themes to emerge from the data. To ensure the trustworthiness of the coding and interpretations of the data, every fifth transcript was coded in duplicate, and any discrepancies were discussed with a third researcher to reach consensus. Coding of transcripts continued until saturation.

The project team at the Department of Public Health, University of Liverpool, UK, undertook all data analyses. The interview transcripts were analysed using the ‘Framework approach’ [15] which follows five pre-defined stages: (1) familiarisation, (2) identification of a thematic framework, (3) indexing, (4) charting and (5) mapping and interpretation. Through an in-depth exploration of the emergent findings, the analysis then identified key themes and linkages between them [16].

### Triangulation, synthesis of mapping exercise and interviews with key informants

Findings from the interviews were cross checked with the information contained in the policy database. Where applicable, the qualitative information from the interviews was used to illustrate national nutrition policy actions in terms of the "4Ps" framework. The interview data also provided additional information in terms of the perceived effectiveness and cost effectiveness of policy actions and the future actions required to reduce cardiovascular disease.

Further information about the methodology can be found in Additional file 2.

### Ethics statement

The Institute of Psychology, Health and Society Research Ethics Committee at the University of Liverpool, England granted ethical approval for the study. Written consent was obtained from participants taking part in face to face interviews. Verbal consent was obtained from participants interviewed either by telephone or Skype. Verbal consent was transcribed and documented. For all participants consent was obtained before the interview commenced. The ethics committee approved both methods of consent used.

This study adhered to the RATS guidelines for reporting qualitative studies.

## Results

We approached 120 experts for an interview, of which 71 agreed (response rate 59%). Of the 71 respondents, 59 were experts in food and nutrition, 6 were policy makers and 6 were senior policy makers. Approximately 60% of the participants were employed in Government Ministries or Universities, about a quarter of the participants represented NGOs and about one tenth of the participants had dual roles, actively participating in or leading NGOs as well as formal government employment.

Tables 2, 3, 4 and 5 provide more detailed information on the findings presented in the results section. The text boxes provide examples of comments made by interview participants (with their country of origin and number assigned to respondent in brackets). The full text of the qualitiative data can be found in Additional file 3.

**Table 2:** **Mapping of existing and planned policy actions within 30 European countries**

	Price			Product	Place			Promotion			
Country	Legislation/Regulation	Taxation	Subsidies ^a^	Reformulation (V/M)	Schools	Workplace	Other settings	Labelling (V/M)	Guidelines ^b^	Advertising controls to children (V/M)	Campaigns
**Austria**	**√**	**X**	**X**	**V/M**	**√**	**√**	**X**	**V**	**√**	**V/M**	**√**
**Belgium**	**√**	**X**	**X**	**V/M**	**√**	**√**	**X**	**V**	**√**	**M**	**√**
**Bulgaria**	**X**	**X**	**√**	**V/M**	**M**	**X**	**X**	**X**	**√**	**X**	**√**
**Cyprus**	**X**	**X**	**X**	**V**	**√**	**X**	**X**	**√**	**√**	**V**	**√**
**Czech Republic**	**X**	**X**	**X**	**V**	**X**	**X**	**X**	**V**	**√**	**V**	**√**
**Denmark**	**√**	**√**	**X**	**V/M**	**V**	**V**	**V**	**V**	**√**	**M**	**√**
**Estonia**	**√**	**X**	**X**	**V**	**M**	**V**	**X**	**V**	**√**	**V**	**√**
**Finland**	**√**	**√**	**X**	**V/M**	**M**	**M**	**X**	**M**	**√**	**V**	**√**
**France**	**√**	**X**	**X**	**V**	**√**	**√**	**X**	**M**	**√**	**V**	**√**
**Germany**	**X**	**X**	**X**	**V**	**√**	**√**	**√**	**V**	**√**	**V**	**√**
**Greece**	**√**	**X**	**X**	**V**	**M**	**X**	**√**	**X**	**√**	**V**	**√**
**Hungary**	**√**	**√**	**X**	**V/M**	**√**	**√**	**X**	**V**	**√**	**V/M**	**√**
**Iceland**	**√**	**X**	**X**	**V**	**X**	**X**	**X**	**M**	**√**	**M**	**√**
**Ireland**	**X**	**X**	**X**	**V**	**X**	**√**	**√**	**V**	**√**	**M**	**√**
**Italy**	**X**	**X**	**X**	**V**	**x**	**√**	**x**	**X**	**√**	**V**	**√**
**Latvia**	**X**	**√**	**X**	**M**	**M**	**X**	**M**	**X**	**√**	**M**	**√**
**Lithuania**	**X**	**X**	**X**	**M**	**M**	**√**	**X**	**V/M**	**√**	**M**	**√**
**Luxembourg**	**X**	**X**	**X**	**O**	**√**	**√**	**X**	**O**	**√**		**√**
**Malta**	**X**	**X**	**X**	**V**	**M**	**√**	**X**	**O**	**√**	**O**	**√**
**Netherlands**	**X**	**X**	**X**	**V/M**	**X**	**√**	**X**	**M**	**√**	**M**	**√**
**Norway**	**√**	**X**	**√**	**X**	**X**	**X**	**X**	**V**	**√**	**M**	
**Poland**	**X**	**X**	**X**	**V**	**√**	**X**	**X**	**V**	**√**	**X**	**√**
**Portugal**	**√**	**√**	**X**	**V/M**	**√**	**X**	**√**	**V**	**√**	**V**	**√**
**Romania**	**√**	**X**	**X**	**V/M**	**M**	**X**	**X**	**V**	**√**	**V**	**√**
**Slovakia**		**√**	**X**	**V/M**	**√**	**X**	**X**	**V**	**√**	**V**	**√**
**Slovenia**	**√**	**X**	**O**	**V/M**	**M**	**√**	**X**	**V**	**√**	**V/M**	**√**
**Spain**	**X**	**X**	**X**	**V**	**√**	**√**	**X**	**M**	**√**	**V**	**√**
**Sweden**	**X**	**X**	**√**	**V**	**M**	**√**	**X**	**V**	**√**	**M**	**√**
**Switzerland**	**√**	**X**	**X**	**M**	**X**	**X**	**X**	**O**	**√**		
**UK**	**√**	**X**	**X**	**V**	**√**	**√**	**X**	**M**	**√**	**M**	**√**

*Data current to end of February 2013.*

Notes for Table 2. ^a^This table does not include information about the EU School Fruit/Milk Schemes or school food subsidies and vending machines in schools. ^b^This includes Food Based Dietary Guidelines as well as other guidelines e.g. Guidelines for healthy nutrition in primary schools or hospitals.

**√** = Yes.

**X** = No.

**O** = Unclear.

**V** = Voluntary.

**M =** Mandatory.

**V/M** = Both (e.g. Mandatory for salt, voluntary for saturated fat).

**Table 3:** **Existing and planned policy actions in relation to salt within 30 European countries**

Country	Action: Salt			
	Price (Legislation/regulation/subsidies)	Product (Reformulation)	Place (Schools, workplace, other settings)	Promotion (Labelling/guidelines/advertising controls/campaigns
**Austria**		Salt Reduction Program "Less Salt is Healthier" ("Weniger Salz ist g’sünder") Joint initiative between Ministry of Health and the Industrial Bakers of Austria. Aims at reducing the salt content in bakery products by 15% by 2015. Dialogue with food industry bread, meat, ready meals.	Guidelines for school catering. Since the beginning of 2012 the implementation of these guidelines take place as part of the initiative "Our School Catering" ("Unser Schulbuffet").	Salt reduction in National Nutrition Plan 2011. Dietary salt target.
**Belgium**	Legislation since 1985. 2% maximum salt content in bread.		Salt Strategy: Stop Salt (self-regulation adopted by food industry, distribution sector, restaurant and catering school sector).	Salt Strategy: Stop Salt Self-regulation adopted by food industry, distribution sector, restaurant and catering school sector.
**Bulgaria**	2009: Ordinance established to reduce salt content of foods in school canteens. For 2011–2012, an updated ordinance includes healthy nutrition and salt reduction in kindergarten school canteens.		Special ordinance for healthy nutrition at schools 2009 and 2011. Food products with a high content of salt are not allowed.	Salt strategy/policy included in the National Food and Nutrition Action Plan 2005–2010. The 2007 National Salt Initiative set a target for consumption of 5 g/day.
**Cyprus**				Dialogue with industry regarding reduction of salt content in bread.
**Czech Republic**		Gradual reduction of sodium levels in dried soups and sauces to 50% of the Guideline Daily Amounts i.e. 1.2 grams of sodium or less.		
**Denmark**				New strategy to reduce population salt intake adopted 2011–12.
**Estonia**		Salt policy included in National Health Plan 2009–2020 and in the National Strategy for CVD 2005-2020.		Industry led discussion.
**Finland**	National legislation on compulsory ‘warning labelling’ of high salt foods since 1980s. Tightened 2009. Upper limit to salt content of eligible products e.g. cheese 1.3%. 2011: Quality criteria to obtain subsidies for meals at university restaurants renewed, contain limits for salt in main meals and all meal components.			Foods that are high in salt are required to carry a "high salt content" warning. A "high salt content" must be labelled, if the salt content is more than 1.3% in bread, 1.8% in sausages, 1.4% in cheese, 2.0% in butter, and 1.7% in breakfast cereals or crisp bread.
**France**		Bakery industry reducing salt in bread. Dietary salt target.		
**Germany**			Recommendations to reduce salt intake are included in all national quality standards for meals in schools, kindergartens, homes for the elderly, canteens at the work place, food on wheels-services.	Nutrition considered a comprehensive approach within the line of the national "In Form" Action Plan. A dialogue with industry will be taken up where considered necessary.
**Greece**	Legal requirement re: max level of salt permitted in bread, tomato juice and tomato concentrates/ purees since 1971. Max level - nutrient profiles that serve as the scientific basis for legislation regarding the list of foods allowed to be sold in school canteens include maximum sodium level requirements. Level of sodium in biscuits: 0.5 g/100 g since 2006.	Hellenic Food Authority working with food manufacturers to reformulate processed products high in salt.	Nutrient profiles that serve as the scientific basis for legislation regarding the list of foods allowed to be sold in school canteens include maximum sodium level requirements.	Salt strategy mentioned in the Action Plan for Implementation of the National Nutrition Policy.
**Hungary**	**Codex Alimentarius Hungaricus** modified salt content for bread and some other bakery products (on dry matter).			Dietary salt target. Salt included in in Hungarian National Nutrition Action Plan 2010–2013.
	**Tax: Act CIII on public health product tax:** salty snacks with salt content exceeding 1 g/100 g and condiments (soup and other powders, artificial seasonings) above 5 g salt /100 g (2011). Max level ‘Nutritional recommendation for mass caterers’ draft proposal for a ministerial decree - Recommendation issued (ministerial decree is in progress).			
**Iceland**		Setting benchmarks for salt reduction, reformulation.		Multilevel awareness raising public campaigns; cooperation with the food industry; and monitoring and evaluation.
**Ireland**				Salt strategy included in Changing Cardiovascular Health, National Cardiovascular Health Policy 2010–2019. Dietary salt target.
**Italy**		July 2009 - Voluntary Agreement between associations of craft bakers and plant bakers and Ministry of health to reduce salt content in some of their products. Ref: P. Strazzullo, G. Cairella, A. Campanozzi, M. Carcea, et al. for the GIRCSI Working Group 1.		National salt reduction initiative since 2008 in line with EU target of 16% Reduction by 2013. Population based strategy for dietary salt intake reduction: Italian initiatives in the European framework Nutrition, Metabolism and Cardiovascular Diseases, 22(3) 2012 161-166.
**Latvia**	Dietary standards in schools, kindergartens, long-term social care institutions and hospitals (2012).		Salt maximum level - Dietary standards in schools, kindergartens, long-term social care institutions and hospitals.	
**Lithuania**	Course of action for Nursery school, Primary and Secondary school children and Foster Home Nutrition, article 17 prohibits confectionary which contains sodium >0,4 g/100 g (2011).		Salt maximum level - Dietary standards in schools, kindergartens, long-term social care institutions and hospitals.	Dietary Salt Target
**Luxembourg**				As of 2008 initiatives discussed with bakers and butchers federations.
**Malta**		Dialogue with industry to reduce salt began in 2010. National salt reduction initiative focuses on bread only by 2012.		Salt mentioned in a Strategy for the Prevention and Control of NCDs in Malta 2010.
**Netherlands**	Bread max. 2.5% salt on dry matter, tightened to max. 2.1% salt on dry matter (1.8% salt in flour). 2013 it will be tightened again to 1.8% salt on dry matter (1.5% in flour).			
**Norway**				Banned all food advertising targeting children aged younger than 12 years since 1990. National salt initiative reduction is linked to the Action Plan on Nutrition (2007–2011), "Recipe for a healthier diet".
**Poland**				Salt reduction included in the National Prevention Programme of Overwieght, Obesity and Non-communcable Disease through Diet and Physical Activity Improvement 2007–2011 and the national Health Programme 2007–2015.Dietary Salt Target Salt initiatives being developed as of 2010. Ministry of Health financed salt reduction programme 2009–2011.
**Portugal**	Tax introduced 2012 for VAT on salty products.	Law adopted August 2009 to set the maximum content of salt in bread.		Law adopted August 2009 to set the maximum content of salt in bread and enact guidelines for the labelling of pre-packaged foods for human consumption, compelling the inclusion of visible data on the relative and absolute quantity of salt on the packaging.
	Voluntary Initiatives with the food industry.	Voluntary Initiatives with the food industry.		
**Romania**	Ministerial Order 1563/2008: Food with salt content above 1.5 g salt/100 g or 0.6 g sodium/100 g not allowed to be sold in schools.	The Ministry of Health and Romalimenta are in the process of signing an agreement for the reformulation of foods with salt. Voluntary reformulation meat and bread products by industry.	Ministerial Order 1563/2008: Food with salt content above 1.5 g salt/100 g or 0.6 g sodium/100 g not allowed to be sold in schools.	
**Slovakia**	National legislation, focus on maximum level of salt in some food categories since 1996. Currently preparing an amendment.	Voluntary by some food business operators.		Salt strategy mentioned in the National Obesity Prevention Programme.
**Slovenia**	Nutritional recommendation for salt content in bread and meat products since 2010.			Discussion with food industry (Chamber of Commerce) started in 2009 with a seminar in April 2010. The National Action Plan for Salt was adopted in July 2010. And a national salt campaign was launched in May 2010 to March 2011. Salt strategy included in National Programme of Food and Nutrition Policy 2005–2010. Salt specific programme.
**Spain**		Voluntary by industry. Agreement with Bakery Confederation regarding salt reduction in bread.		Salt strategy mentioned in the *Strategy for Nutrition, Physical Activity and Prevention of Obesity (NAOS)* (Schafer Elinder and Bollars).
**Sweden**				Banned all food advertising targeting children aged younger than 12 years since 1990.
				Government has dialogue with food industry as of 2010. Salt strategy/policy mentioned in "Healthy Habits and Increased Physical Activity", the basis for an Action Plan.
**Switzerland**		Negotiating with industry to reduce salt in bread and processed foods.The big food manufacturers and retailers made commitments in line with the EU Framework.		Salt strategy 2008 – 2012 included public awareness campaigns and a commitment to work with the food industry.
**UK**	Wales: max level - mandatory requirements for foods vended in hospitals requiring hospital caterers to vend lower salt products (as defined by FSA traffic light labelling criteria).	England, Northern Ireland, Scotland, Wales: Voluntary by industry.	Wales: max level - mandatory requirements for foods vended in hospitals requiring hospital caterers to vend lower salt products (as defined by FSA traffic light labelling criteria).	A voluntary consistent system of front-of-pack food labelling has been introduced: A combination of colour coding and nutritional information is used to show how much fat, salt and sugar and how many calories are in each product.
			As part of the government’s Responsibility Deal, 49 companies/retailers have agreed to provide calorie information on menus and display boards. Although voluntary, the label must follow a standard government model.	Since November 2006, Ofcom, an independent communications regulator in the UK, announced a ban on television advertising of products high in fat, salt or sugar during children’s airtime and around programmes with a disproportionately high child audience.
				England, Northern Ireland, Scotland, Wales:; National Salt reduction strategy.

*Data current to end of February 2013.*

**Table 4:** **Existing and planned policy actions in relation to trans fatty acids**

Country	Action: Trans Fatty Acids (TFAs)			
	Price (Legislation/regulation/subsidies)	Product (Reformulation)	Place (Schools, workplace, other settings)	Promotion (Labelling/guidelines/advertising controls/campaigns
**Austria**	TFAs banned since 2003			
**Belgium**		Voluntary reformulation on sugary foods, fat and TFAs.		Working group formed 13.01.12 to examine Saturated Fat, TFAs, sugar, portion size and fibre in bread flour.
**Denmark**	TFAs banned since 2003			
**Estonia**				TFAs policy included in the National Strategy for the prevention of CVD 2005-2020.
**Germany**				2012: Joint initiative of the German Food Sector and the Ministry of Nutrition, Agriculture and Consumer Protection (BMELV) concerning "Guidelines to minimize TFAs in food": Giving practical recommendations to industry how to further reduce non-ruminant (industrial) TFA in food.
**Hungary**	Draft ministerial decree on the maximum levels of TFAs in foodstuffs.			
	A ministerial decree has been drafted to set up a limit for the trans fatty acid content of foodstuffs being on the market, taking into account the WHO recommendation for daily trans fatty acid intake. The professional consultation of the content of the draft decree is ongoing.			
**Iceland**	TFAs legislation since August 2011. TFAs (less than 2% per 100 g fat).			
**Italy**		Discussions to improve partnership with food industry. Some important results have been reached, like the reduction of trans–fats from sweet products. Some voluntary agreements have been reached with sweet producers regarding elimination of trans-fats.		
**Netherlands**		Voluntary by industry - total fat, TFAs and sugary foods.		
**Spain**		Voluntary by industry		Voluntary by industry TFAs strategy mentioned in the Strategy for Nutrition, Physical Activity and Prevention of Obesity (NAOS) (Schafer Elinder and Bollars).
**Switzerland**	TFAs banned since 2008			
**UK**		Voluntary by industry		

Data current to end of February 2013.

**Table 5:** **Existing and planned policy actions in relation to total fat, saturated fats, sugar and fruit and vegetables**

Country	Action: Total Fat, Saturated Fats (SFs) and sugar			
	Price (Legislation/regulation/subsidies)	Product (Reformulation)	Place (Schools, workplace, other settings)	Promotion (Labelling/guidelines/advertising controls/campaigns
**Austria**			Guidelines for school catering. Since the beginning of 2012 the implementation of these guidelines take place as part of the initiative "Our School Catering" ("Unser Schulbuffet").	
**Belgium**		Voluntary reformulation on sugar, SFs and TFAs.	The provision of free or subsidized fruit and vegetables, as well as a ban on unhealthy food in vending machines at school are only partially enforced, and dealt with at local level.	Working group on SFs, TFAs, sugar, portion size and fibre in bread flour set up 13.01.12.
**Bulgaria**	Special ordinance for healthy nutrition at schools 2009 and 2011, introduced 2011–12. Bulgarian State standard covers milk products, Bulgarian yellow cheese, standards for meat and poultry products including sausage.	Dialogue and some action with meat products, bread/bakery products and soft drinks producers. Actions are all regional or local level.	July 2009 ordinance of the Ministry of Health adopted. Mandatory provision of school cafeteria with vegetables, fruits and other healthy foods, and the restriction of sales of energy-dense and nutrient-poor foods and beverages in school canteens, cafeteria and vending machines. Food products with a high content of fat and sugar are not allowed.	
**Cyprus**			Some efforts have been made to remove unhealthy food and beverages from school vending machines.	
**Denmark**	SF tax on foods with over 2.3% Sat Fat introduced Oct 2011; repealed Nov 2012.		Restaurants must be able to prove that the courses on the menu marked with the "Keyhole" symbol lives up to the expectations of being low in the content of fat, sugar and salt and high in the content of fibre.	
	2009 introduction of the "Spring Package" included an increase of fiscal taxes on confectionary and soft drinks and investigated the possibilities of creating a fiscal tax on SF. Has had levy on candy for 90 years.			
**Estonia**			The National Health Development Plan for Estonia contains specific actions regarding removal of energy-dense nutrient poor foods and beverages in school vending machines.	Discussion regarding total fat, sugar and salt
**Finland**	Agricultural subsidies to encourage dairy farmers move to berry production.	Voluntary action industry led regarding SFs.	Quality of school meals regulated by Ministry of Education and Culture. 2007 recommendations that vending machines should not provide sweets and beverages in schools.	Voluntary action Guidelines on how to include nutritional criteria (such as SFs) in food service procurements were implemented late 2009.
	Tax currently exists for soft drinks, ice cream and chocolate. Discussions under way to increase this tax. Aim of the standing government is to have an agreement on general tax on sugary foods before the year 2013. All food in Finland taxed at 13% despite calls for fruit and vegetables to be taxed less or not at all.			
**France**	A tax on sugar sweetened beverages became effective in January 2012. The tax was set at about 11 euro cents for a 1.5 litre of soda, about 6% of the average price of sodas.	Dialogue with industry regarding reducing fat and sugar in food products.	Vending machines not allowed in school settings since 2005.	Dialogue with industry regarding Included in Second National Nutrition and Health Programme 2006–2010.
			Local level initiatives ensuring a choice of healthy food at the workplace.	
**Greece**			2006: Law on the availability and quality of foods available in school canteens. Vending machines are not allowed in schools.	
			Draft ministerial decree on the nutritional-health provisions in public catering.	
**Hungary**	A "public health tax" adopted in 2012 is applied on the salt, sugar and caffeine content of various categories of ready-to-eat foods, including soft drinks (both sugar- and artificially-sweetened), energy drinks, pre-packaged sugar-sweetened products.		Recommendations for healthy schools buffet options included in government resolution on education.	
			Local level initiatives to ensure a choice of healthy food at the workplace.	
**Iceland**	Regulation 1924/2006 establishes EU-wide rules on the use of specified nutrient content and comparative claims (i.e. levels of fat for a low fat claim). Nutrition claims can only be used on foods defined as "healthy" by a nutrient profile (nutrient profile not yet defined).			
**Ireland**			Recommendations regarding the choice of healthy food at the workplace within the Happy Heart at Work Programme ". Happy Heart at Work Healthy Eating Award (Ended 2012) designed by Irish Heart Foundation to assist employers provide healthy food choices in the workplace. Also a ‘Happy Heart Catering Award’ in the northeast of the country which targets cafes, hotels, pubs etc.	Discussions to develop initiatives to reduce the content of fat and/or sugars in processed foods.
**Italy**		Discussions to improve partnership with food industry and develop initiatives to increase the availability of processed foods with reduced fat/added sugars. Ministry of Health has been encouraging the primary producers and the processing industry to progressively reduce the total content of fat, saturated fats, sugar and added salt in food products. Some voluntary agreements have been reached with the bakery industry (regarding salt reduction), sweet producers (regarding elimination of trans-fats) and retailers (through a national information campaign that encourages fruits and vegetables consumption	The National Plan of Prevention and the regional "Gaining health" schemes endorse several projects to promote healthy nutrition in the workplace.	
**Latvia**	2012: Dietary standards in schools, kindergartens, long-term social care institutions and hospitals. Sausages, frankfurters, dried, smoked, salted meat and fish products, factory made ravioli, frozen manufactured meatballs and fish fingers, etc. are allowed once a week if they contain at least 70% meat or 60% fish; Increased taxes on food high in fat, salt (other than sodium), and sugar nutrients.		2006: Government implemented legislation that prohibited the sale/availability of soft drinks, drinks with added colours, sweeteners, preservatives and caffeine on all school premises. 2012: Dietary standards in schools, kindergartens, long-term social care institutions and hospitals.	
**Lithuania**			2005 restrictions on unhealthy food in school catering, especially vending machines.	
**Luxembourg**			National and local level efforts to remove energy-dense nutrient-poor foods and beverages in school vending machines.	As of 2010 initiatives being discussed but reformulation of fat and sugary foods not a priority at present.
**Malta**			Vending machines not allowed in any public schools and most private schools.	
**Netherlands**		Voluntary by industry - total fat and sugary foods.	Dutch Nutrition Centre has implemented some actions promoting healthy nutrition at work.	
**Norway**	Regulation 1924/2006 establishes EU-wide rules on the use of specified nutrient content and comparative claims (i.e. levels of fat for a low fat claim). Nutrition claims can only be used on foods defined as "healthy" by a nutrient profile (nutrient profile not yet defined).			Banned all food advertising targeting children aged younger than 12 years since 1990.
**Poland**				Government discussing total fat and sugar content of processed food products.
**Portugal**			Encourage the provision of healthy food products in schools.	Developments to increase availability of processed foods with reduced content of total fat and/or sugar through the National Platform Against Obesity.
			Guidelines exist for restaurants as part of Platform Against Obesity. By Nov 2010 86,000 enterprises had implemented the guidelines.	
**Slovakia**	2009 VAT reduced from 16% to 9% for farm products - especially milk, fruit and vegetables. Direct support for fruit and vegetables especially organic and integrated production, which allows for lower prices of fruits and vegetables for consumers.			
**Slovenia**			Food guidelines and legislation 2010. Vending machines banned all primary and secondary schools.	
**Spain**			National legislation proposed 2010 regarding vending machines in schools needs to be adopted by regional governments. July 2010 recommendations and technical criteria for the feeding and food supplies in schools. Free/subsidized F&V schemes being developed.	
**Sweden**			As of 2010 Education Act requires in all schools (kindergarten, primary, secondary) that school meals have to be nutritious. Guidelines from the National Food Administration on planning, producing and serving healthy food at school have also been issued.	Banned all food advertising targeting children aged younger than 12 years since 1990.
**UK**	All unprocessed food stuffs are zero-rated value-added tax. A range of unhealthy foods have standard rated value-added tax.		All food in state schools (voluntary in academies) must meet nutritional standards. Meals must include high-quality meat, poultry or oily fish, at least 2 portions of fruit and vegetables with every meal, bread, other cereals and potatoes. No fizzy drinks, crisps, chocolate or sweets in school meals and vending machines and no more than 2 portions of deep-fried food a week.	A voluntary consistent system of front-of-pack food labelling has been introduced: A combination of colour coding and nutritional information is used to show how much fat, salt and sugar and how many calories are in each product.
			Various workplace initiatives taken to ensure a choice of healthy food.	Since November 2006, Ofcom, an independent communications regulator in the UK, announced a ban on television advertising of products high in fat, salt or sugar during children’s airtime and around programmes with a disproportionately high child audience002E.
			As part of the government’s Responsibility Deal, 49 companies/retailers have agreed to provide calorie information on menus and display boards. Although voluntary, the label must follow a standard government model.	The Change4Life Convenience Stores programme is a partnership between the UK Department of Health and the Association of Convenience Stores to increase the availability of fresh fruit and vegetables in convenience stores in deprived, urban areas in England with poor existing retail access to fresh fruits and vegetables.

*Data current to end of February 2013.*

Table 2 provides an overview of existing and planned policy actions within all the 30 countries. Subsequent tables summarise activities, based upon the "4Ps" framework in relation to specific nutrients: salt, trans fatty acids and total fat, saturated fats, sugar and fruit and vegetables.

### Analysis of food policies across the 30 European countries using the "4Ps" framework

#### Product: *reformulation; elimination or new healthier products*

Activity relating to "Product" primarily focused upon reformulation, especially salt reduction.

### Reformulation: mandatory initiatives

Thirteen countries have legal requirements regarding the maximum *salt* content in certain foods (Belgium, Bulgaria, Finland, Greece, Hungary, Latvia, Lithuania, Netherlands, Portugal, Romania, Slovak Republic, Slovenia and Wales) (Table 3).

*Trans fat bans* exist in Austria, Denmark, Iceland and Switzerland. Denmark was the first country to introduce such legislation in 2003 which strictly regulated the sale of many foods containing trans fats. This was actually preceded by a decade of increasing public and political pressure and stepwise reformulation by industry (Table 4).

Legislation or regulation affecting sugar, fat and fruit and vegetable consumption was uncommon (Only 4 out of the 30 countries). Finland, France and Latvia have legislation affecting sugary products. Latvia has legislation affecting fat and sugary foods, and Slovakia has legislation affecting fruit and vegetables (Table 5).

Many participants commented that the mandatory reformulation of food products was perceived as an effective and cost-effective approach for improving public health nutrition. It was perceived as acceptable to the food industry and the public alike. Food industry profit margins would not be affected and the public would subconsciously be reducing their risk of CVD by eating less salt, sugar and saturated fat in everyday food products (Table 6).

**Table 6:** **Voluntary & mandatory reformulation**

Comment	Source
*"…we need to do something for health related regulations like smoking campaigns, the smoking ban and the same thing is for trans fats or for fat or for sugar or for salt. We need to do something about the regulation because if we leave it to the voluntary, it will be very, very low…"*	*Italy 2*
*"…the reformulation of products will have one of the greatest impacts because if you get reformulation of foods… in Malta we know that people eat a lot of bread so we like get the salt within the bread slowly reduced in the bread they’re gonna eat, and that doesn’t really need behaviour change because they are eating whatever they are finding available. So we reduce like the trans fats within the products which are available and then they are eating less trans fats. So that maybe one of the easiest approaches but we have to tackle industry."*	*Malta 5*
*"No, it [voluntary reformulation] might be effective, but I’m not sure that it is being done in a substantial way. It’s only being done for those food products where the company can use it as an additional unique selling point, to say that they have less fat content than the other company. So it will work for a number of foods but it will not work for the vast majority of foods. Regulating the levels of fat and sugar in foods would be more effective, forcing food companies to do it."*	*Belgium 1*

### Reformulation: voluntary initiatives

Voluntary reformulation of foods by the food industry was common, occurring in 25 of the 30 countries, most commonly for salt (Table 3) (e.g. Austria, Belgium, France, Greece, Italy, Malta, Portugal, Romania, Sweden and the UK) (Tables 2, 3, 4 and 5).

Estonia, France and the Netherlands have voluntary reformulation in relation to sugary foods and total fat (Table 5). For example, in France, there is dialogue with industry regarding the fat and sugar content of certain foods and this was included in the Second National Nutrition and Health Programme 2006–2010 (Table 5).

#### Price: taxes; subsidies and other economic incentives

Price incentives in different European countries targeted various unhealthy nutrients, including salt, sugar and saturated fat. Taxes to promote healthy nutrition (e.g. fruit and vegetables) are currently only used by six countries. Finland, France, Hungary and Latvia have implemented ‘sugar taxes’ on sugary foods and sugar-sweetened beverages, while Portugal is the only country that taxes salty products. Hungary taxes food high in fat (Tables 3, 4 and 5).

From 2011, Finland reinstated taxes on sweets (e.g. candies, chocolate, cocoa-based products, ice cream, ice lollies) that existed until 1999. The existing tax on soft drinks was also increased and its scope was widened to cover further categories of beverages. Discussions are being held to further extend this tax (Table 5). In 2012, the French Government introduced a tax on sugar-sweetened drinks including artificially sweetened drinks (fruit juices with added sugars, water, carbonated drinks containing added sugar) (Table 5). In 2011, Hungary introduced a public health product tax on snacks with a salt content exceeding 1 g/100 g and condiments (soup and other powders, artificial seasonings) above 5 g salt /100 g; plus taxes on soft drinks, pre-packed sweetened products and energy drinks (Table 3).

In October 2011, Denmark was the first country to introduce a tax on saturated fats (meat, cheese, butter, margarine, snacks, etc.) with the intention of decreasing consumption levels by 4% [17]. However, following co-ordinated action by the food industry, the tax was repealed in November 2012 (Table 5).

The majority of interview respondents felt that "Price" incentives such as taxes, legislation and regulation were the most effective options for improving public health nutrition (Table 7).

**Table 7:** **Price: comments on taxes and other economic incentives**

Comment	Source
*"…legislation and regulation. Because with voluntary actions you will get little wins in the short term but not all actors will be moving and with regulation you get one rule for everyone so there is less inequity…"*	*Belgium 2*
*"I think it is necessary to adopt more laws … maybe taxes on, higher taxes on junk food and therefore the healthier food would be cheaper."*	*Czech Republic 3*
*"I think the taxation is very effective. I would go to taxation of the sugar because it is how it is used now and how people drink the soft drinks and eat candies so I would tax it more…"*	*Finland 5*
*"…maybe taxation could be most cost effective, however there is a problem of political costs of such activity. Taxing probably products rich in saturated fats. Then products like sweeties which are high in calories and also which are rich in saturated fat and sugar."*	*Poland 2*

Subsidises for healthy food products were uncommon, apart from the almost universal EU School Fruit Subsidy Scheme. Co-funded by the EU and individual Member States, this voluntary scheme aimed to encourage good eating habits in young people by making fruit and vegetables available to children in schools. In addition, participating Member States were required to set up strategies including educational and awareness-raising initiatives. However, target groups, take-up and implementation of the scheme has varied widely, making comparisons between countries difficult.

*"…if there is taxation or subsidies, there should be legislation and regulation, you can’t divide them. For example for school fruit scheme we need legislation as well. But I think that subsidies might be one possibility just to offer cheaper healthy foods. Or to subsidise to influence farmers to grow fruit." (Estonia 3)*

#### Promotion health education, public information & campaigns, advertising controls, food labelling

**Information and health education** Information campaigns targeted at the general population were widespread (Table 2). The majority focussed upon general healthy eating messages and or campaigns targeted at reducing childhood obesity. Some countries also highlighted specific nutritional topics such as salt (e.g. Belgium, England, Estonia, Ireland, Italy, and Slovenia). However, participants generally perceived such interventions to have limited impact (Table 8).

**Table 8:** **Information and health education**

Comment	Source
*"…they (the Government) always like information and communication campaigns because they prefer to have visibility. That’s also sometimes a problem because in the first place they want some concrete visible action instead of structural changes but in fact it is always a combination of those options in fact."*	*Belgium 2*
*"Certainly the Government would be most willing to do, would be giving some money for education and information campaigns, which in my opinion would be the least effective."*	*Germany 1*
*"I think we can forget education and information campaigns. OK they are good but they are not going to be very effective. So I think the most effective points would be legislation, regulation and taxation."*	*Iceland 1*

Many countries include nutrition education as a mandatory part of the school curriculum (Austria, Bulgaria, Denmark, Estonia, Finland, France, Germany, Ireland, Latvia, Lithuania, Portugal, Slovakia, Slovenia, Sweden, and UK) and most are also actively improving the nutritional value of foods available in schools.

### Labelling

Food labelling of nutritional composition was common (Table 2). 21 countries had some form of labelling; however, presentation and information varied widely. Since 2009 it has been mandatory in Portugal to list the salt content of food and the sodium content in bread is restricted to a maximum of 14 g/kg (Table 3). Under current EU regulations, nutrition labelling is optional, but becomes compulsory if a nutrition/health claim is made on the label. Some countries have adopted nutritional logos. For example, Sweden, Norway, Denmark and Iceland adopted the ‘Keyhole’ scheme as a joint nutrition label. This is a voluntary scheme for food producers, but products labelled with the symbol must conform to nutritional regulations in different food groups (i.e. fat, saturated fat, salt, sugar and fibre). Similar schemes exist in other countries, for example, in Finland ("healthy heart" logo) and the Netherlands ("choices" logo).

Participants perceived labelling as being effective; but felt however, that nutritional information labelling needs to be easy to identify (i.e. front of pack) and straightforward to read and interpret (Table 9).

**Table 9:** **Labelling**

Comment	Source
*"…We need the information but we have many people who don’t read the labels and that’s why we need the labelling on the front of packages and the symbols are important…"*	*Finland 1*
*"So sometimes you have to have both; not only legislation but also try to have information and education and also try to work with parents and teachers and so on. It’s also important to help with labelling because they do not often know how to read the labelling…"*	*Portugal 2*
*"I think if you’re going to provide people with that sort of information it should be understandable and usable and appropriate."*	*England 4*

### Dialogue recommendations and guidelines

Dialogue, recommendations and guidelines are often an early part of the policy process and are widespread (Table 2).

### Marketing of foods high in fat, salt and sugar to children

Although many of the 30 countries were self-regulating, 12 countries had mandatory regulations against marketing to children (Austria, Belgium, Denmark, Hungary, Iceland, Ireland, Latvia, Lithuania, Netherlands, Slovenia, Sweden, and UK). Only a few had regulations for advertising in schools, while many had general regulations for advertising to children but which were not food-specific. Sweden had banned any advertising targeted at children under 12 but because of EU legislation, the ban only covered broadcasts originating in Sweden. In 2011, Iceland introduced a new media law banning adverts adjacent to programmes aimed at children under 12 years as well as provisions regarding commercial communications and teleshopping (Table 2).

Interviewees perceived mandatory measures around marketing of foods high in fat, salt and sugar as clearly being more effective than self regulation (Table 10).

**Table 10:** **Marketing to children**

Comment	Source
*"Stronger control over marketing of unhealthy foods to children, I would certainly put that high as a priority. It’s controllable, it’s achievable, I’d actually possibly put that up there as perhaps the most achievable. It clearly can be done through control over advertising."*	*England 3*
*"Like in advertising, a consensus paper has been written with the food industry about advertising… it has been until now difficult to legislate so we have worked in terms of consensus. But we will try in the future, when it will be possible to have some legislation…"*	*Portugal 2*

#### Place schools and workplaces

Place interventions aim to modify food quality or availability in specific settings. The majority were situated in schools and, to a lesser extent, workplaces (Table 2). Interventions primarily focused on the removal of vending machines, or replacing the contents of vending machines (now offering healthy snacks) , and legislation, regulation or recommendations on food offered in canteens. Many countries are actively improving the nutritional value of foods available in schools.

Participants felt that interventions targeted in school settings or preschool (kindergarten) settings were effective (Table 11).

**Table 11:** **Schools**

Comment	Source
*"… I think nutritional recommendations for school canteens and it is necessary to adopt in law I think. Not only recommendations but law for nutritional content of lunches. And to ban some content of vending machines in schools."*	*Czech Republic 3*
*"But we will try in the future, when it will be possible to have some legislation. We have done that in the schools, in the schools it is not possible to have the fat in food; it is not possible to sell it. And sugar is not possible to sell…that is a regulation for schools."*	*Portugal 2*

### Exemplary food policies and future aspirations

All 71 interviewees were also asked about exemplary food policies and future policy options. These topic experts and policy makers across the 14 countries consistently perceived legislative and regulatory approaches as being generally more effective at improving public health nutrition than voluntary approaches, or information or education campaigns.

### Perceived cost-effectiveness of regulation versus voluntary measures

Participants across all 14 European countries also perceived regulatory measures to be more cost-effective than voluntary measures. The most popular modifiable dietary risk factors to focus upon were salt, trans fat and saturated fat (England, Finland, Germany, Greece, Iceland, Ireland, Portugal, Slovenia). Taxation was highlighted by participants in Czech Republic, Germany, Iceland, Ireland, Poland and Slovenia.

### Future requirements to improve public health nutrition

Participants in thirteen of the 14 European countries perceived legislation and regulation as necessary for improving public health nutrition. Legislation specifically relating to reformulation was identified as necessary by experts in Belgium, England, Estonia, Finland, Germany, Greece, Italy, Portugal and Slovenia. Taxation was also deemed important by participants in Belgium, Czech Republic, England, Finland, Greece, Iceland, Ireland, Poland, Portugal and Slovenia.

## Discussion

### Main findings

The 30 Western and Central European countries studied are at very different stages of addressing healthy diet strategies. For example, countries such as Finland, Hungary and Portugal all have legislation and taxation to improve public health nutrition, whereas Cyprus, Germany and Lithuania do not have such strategies in place. Dialogue, recommendations and guidelines are widespread, but represent an early and uncontroversial part of the policy process. Likewise information and education campaigns which are widespread and include campaigns for the general population, and more targeted campaigns in schools, the workplace and within communities. Many include general healthy eating messages, while some countries also highlighted specific nutritional topics such as salt or fruit and vegetables. However, many expert respondents considered such "downstream" interventions to have limited effectiveness, correctly reflecting published evaluations [11, 18, 19].

Conversely, taxation or regulation is still uncommon. Though many of our respondents correctly perceived these "upstream" interventions as being much more effective and powerful, these are also seen as politically more challenging [11, 18, 19].

New EU legislation has introduced mandatory "back of pack" nutrition labelling and countries need to adopt this by 2016 [20]. However, ‘front-of-pack’ nutrition labelling remains voluntary [21]. Some countries require more detailed information about the nutritional value of foods. However, presentation and the information provided vary widely. Some key informants correctly emphasised that although consumers may look at nutrition labelling, they may remain confused by the information presented.

Mandatory reformulation of products to reduce salt and saturated fat remain uncommon, even though they are generally agreed to be more effective by our policy makers, and by researchers [19, 22]. Barely a dozen European countries have regulations regarding the maximum *salt* content in certain foods.

Food taxes are currently used effectively in a few, notable countries for sugar (Finland, France, Hungary, Latvia) or salt (Portugal) The brief Danish Fat Tax experiment in 2011/12 was unsuccessful but has perhaps provided useful lessons for countries considering the future implementation of "fat taxes" [23].

Advertising food and beverages can powerfully affect children’s food choices and food intake [24]. Some governments have therefore agreed voluntary schemes with large food and beverage manufacturers to try and limit the marketing of these products to children. However, the recent EU media directive merely encourages governments to encourage media service providers to develop codes of conduct. Furthermore, our topic experts were sceptical about the effectiveness of voluntary codes, echoing previous critiques [24, 25].

### Comparisons with other studies

This work provides a 2012 "snap-shot" and analysis of diverse policy actions relating to public health nutrition across Europe. It complements and builds on previous studies. In particular, it extends the WHO 8 countries study, and the PORGROW study (9 countries) and EuroHeart I study (16 countries), [5, 7, 8]. It also provides further detail of recent promising interventions, notably the percieved effectiveness of all policies including taxation and regulation. It thus contrasts with the Eatwell project (partnered with industry), which paradoxically emphasised voluntary approaches. Our findings are also reassuringly consistent with those in the WHO NOPA website (NOPA) [10].

Voluntary approaches were generally viewed sceptically. The European Platform for Action on Diet, Physical Activity and Health [26] is a European Commission led forum for European-level organisations, the food industry and consumer protection NGOs. The Platform aimed to provide examples of coordinated action by different parts of society that will encourage national, regional or local initiatives across Europe. However, a recent evaluation has queried its effectiveness [18].

The recent PHEIAC external evaluation of the Strategy for Europe on Nutrition, Overweight and Obesity [11] was comprehensive and robust, noting that progress within and between countries has been "uneven". Furthermore, most of the actions taken thus far have been "soft", relying mainly on providing information, limited schools interventions or voluntary actions by the food industry [11]. Our findings endorse these criticisms.

It is gratifying to see that our key observations have been replicated by others. Most notably the multi-country review and survey of policymakers 2014 [27] and NOURISHING [28], the World Cancer Research Fund International’s policy framework to promote healthy eating. The former surveyed policymakers, from legislative and executive branches of government, in 11 countries – Brazil, Bulgaria, Canada, Denmark, England, France, Germany, Italy, Mexico, Spain and the United States. They found policymakers’ perceptions of national policy and knowledge of policies and the impact of different approaches differed, both in terms of whether they are a good course of action and whether they are currently in place and effective. NOURISHING have found that many countries have taken food policy actions to address obesity and NCDs. Many more policies have been implemented which remain unreported or unknown. However, overall progress is disproportionately low compared to the size of the burden of non-communicable diseases and the challenges of unhealthy food environments and diets.

### Strengths and limitations

Our project has many strengths. It represents the most up to date and comprehensive mapping exercise detailing food policies across 30 countries in Western and Central Europe. The "4Ps" framework of Product, Price, Promotion and Place offers a potentially useful tool to help create order out of the "policy cacophony" [3].

In 14 diverse countries, detailed policy analysis, visits and interviews with a diverse range of local experts and stakeholders permitted verification and triangulation. The qualitative interviews enabled a more in-depth examination of the findings from the mapping exercise, and allowed verification of current and future policy actions within countries. The interviews also provided rich detail around perceptions of the most effective policies, the extent of their implementation, and the progress towards specific national targets.

*This project also has clear limitations****.*** Firstly, our data may not be complete, particularly in the 16 countries where in-depth interviews were not undertaken. However, most of these countries were previously examined in the EuroHeart 1 or WHO studies, thus major omissions in terms of policies are unlikely. Secondly, there was a disproportionate number of experts in food and nutrition interviewed. A number of additional policy makers were approached but declined, many stating that they were too busy to take part, or that they were unwilling to express their personal views. Thirdly, our study provides only a snapshot of activities up until 2012. We recognise that developments are on-going and will certainly require regular monitoring and updating (ideally in collaboration with colleagues from WHO and other organisations). Fourthly, because we guaranteed anonymity to our respondents, this might limit the details of organisation and hence context. Fourthly, the category "promotion" is uncomfortably broad ranging from media campaigns down to to individual health education. This merits further work. Finally, during the developmental phase, we considered various candidate frameworks, such as the "3As" model used in tobacco control (Affordability, Acceptability, and Accessibility), and Chow et al. "Environmental influences" approach (Policy/Legislation; Public Information; Societal norms; and Neighbourhood environments) [29]. The "4Ps" framework is not flawless. It has no formal hierarchy and possibly provides an arbitrary categorisation of a continuum of policy actions. However, it appears to perform well in practice, and has withstood criticism since first publication in 2011 [14]. This simple framework offers a logical way to categorise different actions and potentially assists to create some order out of the complexity of policy actions.

### Implications for future public health nutrition policies

Encouragingly, the majority of European countries are actively engaged in activities to improve their public health nutrition and decrease the intake of junk food and sugary drinks. Countries demonstrating notable progress include Finland, France, Hungary, Iceland, Portugal and perhaps the UK. Furthermore, looking now beyond Europe, exemplary policies have been successfully implemented to substantially reduce salt, trans fats and refined sugars and increase the consumption of fruit, vegetables and other wholefood in select countries around the world. Thus, exemplar policies for *salt reduction* might highlight not only Finland, Hungary, Iceland, Portugal and the UK, but also Argentina, Turkey, Japan and Fiji [30]. Likewise, exemplary countries for *industrial transfats* elimination or reduction might include not only Denmark, Switzerland, Austria, and Iceland, but also the USA and Korea [31].

## Conclusions

### Implications for future research

Public health nutrition policies in Europe represent a complex, dynamic and rapidly changing arena. The diverse public health nutrition activities across 30 European countries might initially appear complex and bewildering. However, the "4Ps" framework offers a potentially structured and comprehensive categorisation of these diverse interventions. We therefore now propose future work to further develop the "4Ps" framework, then identify and evaluate population-based policy actions carried out across the entire WHO European Region (53 countries).

However, there is clearly no room for complacency. The coverage and implementation of existing nutrition policies remains patchy and variable, a "smorgasbord" rather than a symphony. It is quite logical that countries develop approaches to promote healthy diet that fit the local and cultural situation. This will ultimately involve setting priorities and making choices with a wide range of stakeholders. Yet, increasing evidence suggests that legislation, regulation and taxation will have the greatest impact upon populations in terms of reducing many NCDs and obesity.

Mandatory approaches are clearly more effective than voluntary schemes. Yet most European countries fall well short in their use of the most effective and powerful "upstream" interventions: legislation, regulation, taxation and subsidies. Based upon this premise we recommend the following actions to be considered at the European and national level:

-  Mandatory reformulation of products to reduce salt, saturated fat and trans fats.
-  Increased availability and quality of healthy foods available in school canteens and vending machines.
-  Further salt reduction targets and elimination of industrial trans fats.
-  Restriction of marketing/advertising of junk foods and sugary drinks to children.
-  Duties on sugary drinks and subsidies for fruit, vegetables and other healthy options.

## Electronic supplementary material

Additional file 1: **Study Protocol.** (PDF 480 KB)

Additional file 2: **Methodology.** (PDF 428 KB)

Additional file 3: **Qualitative Interview Findings.** (PDF 1 MB)
