# Supplementary material for: Smorgasbord or symphony? Assessing public health nutrition policies across 30 European countries using a novel framework
Source: BMC Public Health. 2014 Nov 21;14:1195. doi: 10.1186/1471-2458-14-1195 (PMC4251675; doi:10.1186/1471-2458-14-1195)
Supplement: Supplementary file 1 — Additional file 1: Study Protocol. (PDF 480 KB) [file 12889_2014_7324_MOESM1_ESM.pdf]

Additional file 1

EUROHEART II Protocol: **Identifying the most effective and cost effective CVD prevention policies**

BACKGROUND

Cardio vascular disease (CVD) and non-communicable disease (NCD) policy and commissioning decisions are based on a complex process. Current evidence is just one of many contributory factors. EuroHeart1 provided a valuable overview of policies, plans, legislation on public health, CVD, CHD, stroke, hypertension, obesity, diabetes, alcohol, food, tobacco, stress, inequalities in 16 European countries (Table 1 shows the countries included in EuroHeart 1 and countries to be included in EuroHeart 2). They found, amongst other things: Every country reported some type of legislation covering public health, tobacco control and food. Legislation for other issues related to diseases and lifestyle risk factors associated with cardiovascular diseases were less common; programmes to address issues relating to tobacco, public health, physical activity, coronary heart disease and food were most frequently reported across countries; it was difficult to identify budgets allocated for policy/programme implementation, and in most instances, data available around funding was incomplete; most countries reported some type of cardiovascular health and/or cardiovascular disease prevention targets. The report recommended: an assessment of the effectiveness of different cardiovascular health promotion and cardiovascular disease prevention actions or interventions; An in-depth comparison of well-developed cardiovascular policies, their resources, implementation and results in a selected number of countries; a review of the policy literature, surveys for other relevant policies and a policy content analysis.

TABLE 1

EUROHEART I INCLUDED COUNTRIES (16)	EUROHEART II COUNTRIES (30 plus interviews in 14)
Belgium	Austria*
Denmark	Belgium
Estonia	Bulgaria*
Finland	Cyprus*
France	Czech Republic*
Germany	Denmark
Greece	Estonia
Hungary	Finland
Iceland	France
Ireland	Germany
Italy	Greece
Netherlands	Hungary
Norway	Iceland
Slovakia	Ireland
Slovenia	Italy
UK	Latvia*
	Lithuania*

	Luxembourg*
	Malta*
	Netherlands
	Norway
	Poland*
	Portugal*
	Romania*
	Slovakia
	Slovenia
	Spain*
	Sweden*
	Switzerland*
	UK (England & Wales)

*Denotes additional countries included in EuroHeart II

We will now build on these excellent foundations, to:

- review the policy literature and other relevant policies and perform a policy content analysis
- systematically interview policy-makers and thought-leaders to elicit their views on a very wide range of possible strategies and policy options covering the entire CVD and NCD prevention spectrum in 14 countries (Belgium, Czech Republic, England, Estonia, Finland, Germany, Greece, Iceland, Ireland, Italy, Malta, Poland, Portugal, Slovenia)
- Develop a system to categorise and compare these CVD and NCD policies resources, implementation and results
- Systematically analyse the data to identify the most effective and cost effective CVD and NCD prevention policies. Specifically whether falls in major risk factors and CVD deaths have been greater in the EU countries with a larger number of effective national policies.

SPECIFIC OBJECTIVE: Identifying the most effective and cost effective CVD prevention policies

DESCRIPTION OF THE OBJECTIVE

- Elicit the views of EU policy-makers and thought-leaders on a wide range of possible strategies and policy options for CVD and other NCD prevention;
- Compare EU countries specific cardiovascular policies, their resources, implementation and results;
- Review their policies and other relevant literature
- Develop a framework to categorise CVD policies;

Definition of a policy for the purpose of the study: laws, plans, and actions that have been made and implemented by government to achieve societal goals. The policy is made in the "public's" name, and implemented by public and private actors.

Key Question: What are the key features that made implemented policies effective in the included European countries?

METHODS

Definition of prevention policies

For the purpose of this study, policies will be those relating to CVD and other NCD prevention in relation to food . Policies and policy documents are defined as written documents that contain strategies and priorities, with defined goals and objectives and are issued by a public administration.

Collection of the documents

Policy documents will be obtained from the 30 diverse EU countries.

Any relevant national policy documents addressing CVD/NCD prevention will be sought from the areas of health/public health/health promotion/disease prevention/non-communicable diseases. A combination of methods will be employed including an internet-based search of documents on the web sites of: national ministries of health, public health, environment, education, health promotion agencies such as national and international agencies; local and subnational initiatives. Furthermore, a search will be conducted through the Google search engine using the following key words: CVD prevention, NCD prevention, policy, coronary heart disease prevention, guidelines, strategy, programmes, action plan, national programme, and food. In addition, relevant material available at the WHO European Centre for Health Policy (Brussels) will be reviewed.

An overview of the results of the search findings up to this date will be distributed together with a) an initial call for further contributions from key informants and colleagues from the identified countries and b) Key informants will be invited to comment on draft findings and identify any major gaps. This will be done either via email or a focus group, during interviews, or using a questionnaire.

Inclusion Criteria

The inclusion criteria will be policy documents covering cardiovascular disease prevention policies or chronic disease in relation to food (e.g. food labelling, legislation on food fat, sugar and salt content etc.) and health focussed taxation or subsidies.
Policy documents will include: National Acts, Laws, Legislation, Ministerial Decrees (or equivalent); National policies/strategies or plans; and policies/strategies or plans in preparation.

Analysis

Only policy documents available in English will be included in the analysis. If more than one version exists, only the most recent one will be included. Identified policies which were

implemented will be included. Policies not implemented will be excluded. Included documents will be summarised as follows: the key goals/targets/outcomes of the policy.

SYSTEMATIC INTERVIEWS WITH KEY INFORMANTS

Interviews with key informants from 14 of the diverse EU countries identified (Belgium, Czech Republic, England, Estonia, Finland, Germany, Greece, Iceland, Ireland, Italy, Malta, Poland, Portugal, Slovenia) will be conducted simultaneously with the review of the policy literature and policy documents. The purpose of the structured interviews are to elicit views on a very wide range of possible strategies and policy options covering the entire CVD/NCD prevention spectrum.

Key informants will be identified via our contacts and partners in the 14 included EU countries (this will include the European Heart Network – who have links with Heart Foundations across Europe; and European Society of Cardiology who have links with societies across various European countries). Key informants will include key national policy makers, commissioners, civil servants, experts in ministries of health and other organizations and members of NGOs .

The interview will present participants with the CVD/NCD prevention policies in relation to food identified in their country. Participants will initially be asked if there are any additional relevant CVD/NCD prevention policies for food. Participants will then be asked to assess the identified policies in relation to:

a) Policy development and consultation (involvement of different sectors in the preparation and implementation of the policy – who involved; appropriateness of sectors involved)

b) Date/Year when policy was published and implemented, and extent to which policy was implemented

c) Timeframe (clear timeframe specified for the implementation of the policy, including publication implementation milestones, appropriateness and effectiveness)

d) Goals and targets and outcomes (realistic and specific in terms of population groups/organisations and time periods)

e) Legal status (legally binding or nonbinding; formally adopted by government or not)

f) Enforcement and commitment to policy implementation (effectively enforced and by whom)

g) Impact on target population/organisations (success of policy in terms of impact and change)

h) Budget allocation to policy implementation (specific budget allocated to implement the policy, was budget adequate for policy implementation requirements)

i) Surveillance and monitoring (system implemented to measure implementation and effectiveness of surveillance or monitoring system)

j) Evaluation (development or continuations of an evaluation on the implementation and results of the policy and effectiveness of evaluation process)

k) Effectiveness (which policies most effective and why)

Adapted from: Daugbjerg S.B, Kahlmeier S, Racioppi F, Martin-Diener E, Martin B, Oja P, & Bull F. Promotion of Physical Activity in the European Region: Content Analysis of 27 National Policy Documents Journal of Physical Activity and Health, 2009, 6, 805-817

EXPECTED OUTCOME

Results will inform policy-makers and thought-leaders at national and EU level about

- The relationship between effective national CVD/NCD prevention policies and subsequent falls in major risk factors and CVD deaths
- the most effective and cost effective current CVD/NCD prevention policies
- options for future strategies to prevent CVD and other major chronic diseases

BIBLIOGRAPHY

Hunter D. J. & Marks L *Decision-making processes for effective policy implementation* NICE Guidance http://www.nice.org.uk/niceMedia/pdf/SemRef_Decision_Hunter.pdf

Daugbjerg S.B, Kahlmeier S, Racioppi F, Martin-Diener E, Martin B, Oja P, & Bull F. Promotion of Physical Activity in the European Region: Content Analysis of 27 National Policy Documents *Journal of Physical Activity and Health,* 2009, 6, 805-817

Critchley J et al. (protocol) *MEDiterranean studies of Cardiovascular disease and Hyperglycaemia: analytical Modelling of Population Socio-economic transitions.* 2008 Funder: European Commission)

Andrew D Oxman, Arild Bjørndal, Francisco Becerra-Posada, et al. A framework for mandatory impact evaluation to ensure well informed public policy decisions *Lancet* 2010; 375: 427–31

Anna Ritsatakis and Péter Makara *Gaining health: Analysis of policy development in European countries for tackling noncommunicable diseases* World Health Organisation 2009

Gill Cowburn, Prachi Bhatnagar, Susanne Løgstrup *Cardiovascular disease prevention in Europe - the unfinished agenda EuroHeart work package 5: National plans, policies and measures impacting on cardiovascular health promotion and cardiovascular disease prevention* European Union Public Health Programme September 2009

World Health Organisation *The Stepwise framework for effective prevention and control of chronic diseases* WHO Guideline. Preventing choric diseases: A vital investment: WHO global report 2005
