# Supplementary material for: Smorgasbord or symphony? Assessing public health nutrition policies across 30 European countries using a novel framework
Source: BMC Public Health. 2014 Nov 21;14:1195. doi: 10.1186/1471-2458-14-1195 (PMC4251675; doi:10.1186/1471-2458-14-1195)
Supplement: Supplementary file 2 — Additional file 2: Methodology. (PDF 428 KB) [file 12889_2014_7324_MOESM2_ESM.pdf]

Additional file 2

Methodology

Study design

This is a comparative, mixed methods study (quantitative and qualitative), that provides an overview of the current situation across Europe regarding CVD prevention nutrition policies and their effectiveness.

An advisory group was established and met on a monthly basis for the duration of work package 5. Group membership comprised the project team together with academics with expertise in quantitative and qualitative methodology, food policy and public health policy analysis (Appendix 1). The group advised upon the design and development of data collection tools, project progress, analysis and reporting of findings and dissemination.

Conceptual framework

In order to maintain a coherent approach to the work package, a conceptual framework was developed. We looked at a wide range of candidate frameworks, as well as developing some from scratch. The tobacco control approach appeared useful (3As: Affordability, Acceptability, Accessibility). However, after extensive piloting and discussions, we agreed that the most practical and coherent approach was that used by EHN in "Diet, physical activity and cardiovascular disease prevention in Europe" (European Heart Network, November 2011) which is based on the traditional marketing "4Ps" approach: Price, Product, Promotion & Place. Marketers use this model, also known as the "marketing mix", to assess how well products match their target market by considering factors to do with price, product, place and promotion. The EHN publication and its "4Ps" framework are used in the implementation of this work package and work package 7 of EuroHeart II.

Box 1: The "4Ps" marketing mix

Price: taxes; subsidies; or other economic incentives

Product: reformulation; or new products

Place: schools, workplaces & community settings

Promotion: restricting marketing to children and adults (advertising controls); nutritional food labelling; nutritional information on menus; public information campaigns; and health education ("Multi-component interventions" might involve a combination of several approaches)

Literature review

We conducted rapid scoping reviews of the effectiveness of population-wide nutrition policy interventions. We searched six electronic databases. Recent systematic reviews and primary studies provided the main sources of data. All items were assessed systematically for inclusion. Reference lists were screened and key informants identified additional evidence. The literature was assessed and categorised using the "4Ps" framework explained above. Interventions that involved a combination of these approaches were included under a 5th heading: "Multi-component interventions".

The studies included were too heterogeneous to combine quantitatively in a meta-analysis, so they were synthesised as narrative reviews. Individual reviews were produced for salt; fats (including trans fats, saturated fats and total fats); fruit and vegetables; and generic healthy eating initiatives (i.e. those that did not focus specifically on one nutrient category) (Appendix 2).

Interviews with key informants

Ethical approval for the interviews was granted by the Institute of Psychology, Health and Society Research Ethics Committee at the University of Liverpool in July 2011. Policy-makers, thought-leaders and others active in the field of public health nutrition at the national level were interviewed in all EuroHeart II WP5 countries, in order to elicit their views on a wide range of possible strategies and policy options covering the entire spectrum of public health nutrition focused on CVD prevention.

Questions were developed and piloted with key experts in England. This ensured the inclusion of relevant questions which would provide a comprehensive overview of current and potential future national nutrition policy actions within the 14 WP5 countries. Participants were identified through various sources, for example, the European Heart Network and within European countries via national Heart Foundations. Key informants were also identified via published literature, the internet and the 'snowballing technique' by means of expert colleagues. Potential participants were contacted by email explaining the EuroHeart II project and requesting their participation as experts in public health nutrition policies in their country. Interviews were conducted with participants in all countries. Participants included high level policy makers and experts in the field from a diverse range of institutions, for example, Ministries of Health, Education, Social Welfare/Affairs; Chief Executives of large health statistics organisations; National Institutes of Public Health; Universities; National Nutrition Councils and Heart Foundations; Obesity and Diabetes Societies; Consumer Organisations; CINDI Programme officials; Preventive Cardiologists; and Food and Veterinary Authorities. Over half the participants were employed in Government Ministries or Universities (approximately 60%);

about a quarter of the participants represented NGOs and about one in ten of the participants had dual roles, actively participating in or leading NGOs as well as formal government employment. Prior to interviews, participants were emailed an information sheet, a consent form, the interview questions and a summary of public health nutrition policies and related initiatives in their country (see Appendices). The latter two enabled familiarity with the interview content and format. The interviews were conducted either in person, by telephone or Skype. All interviews except one were digitally recorded. The interviews typically lasted between 45 minutes to one hour. In total 71interviews were conducted. The interviews were transcribed and entered into NVIVO software, to aid analysis.

Table 1: Number of key informant interviews across 14 countries

Country	Number of interviews
Belgium	4
Czech Republic	4
England (pilot)	7
Estonia	4
Finland	6
Germany	4
Greece	4
Ireland	4
Iceland	8
Malta	5
Italy	6
Poland	4
Portugal	4
Slovenia	6
Europe-wide perspective	1
**Total**	**71**

Data analysis

The project team at the Department of Public Health, University of Liverpool, UK, undertook all data analyses. The interview transcripts were analysed using the 'Framework approach', which is particularly useful for applied policy research (Pope et al 2000, Ritchie, Spencer and O'Connor 2003). It involves a systematic process of sifting the data, charting and sorting material according to key themes and issues. A hierarchical thematic framework of main themes was developed, subdivided by a succession of related subtopics. Data were classified and organised according to the themes, concepts and emergent categories. Data were charted by completing a matrix or table where each case (individual participant) has its own row, and columns represent the subtopics. Cells contain relevant summaries from the data set. The charts were then used to examine and interpret the data for patterns and connections.

The key steps in the Framework approach are:

1. Familiarization

The researcher gains an overview of the data and becomes familiar with the range, depth and diversity of data gathered. Key ideas and recurrent themes are listed.

2. Identifying a thematic framework

Key issues, concepts and themes are examined, referenced and developed further into a thematic framework. The framework draws upon:

- a priori issues (those informed by the original research aims and introduced into the interviews via the topic guide)
- emergent issues, raised by the participants themselves
- analytical themes arising from the recurrence or patterning of particular views or experiences

The framework is further refined as it is applied to transcripts.

3. Indexing

This is the process whereby the thematic framework is systematically applied to the textual data. All data are read and annotated according to the thematic framework. Indexing references are recorded on the margins of each transcript that then links back to the framework.

4. Charting

Once the thematic framework is applied to individual transcripts, the picture of the data as a whole is built up. This is done by 'lifting' data from their original context and rearranging them according to the appropriate thematic reference. Charts can then be devised with headings and sub-headings that are drawn from a range of sources, including the thematic framework itself; from a priori research questions; or according to considerations about how best to write up the study.

5. Mapping and interpretation

Finally, when all the data have been sifted and charted according to core themes, key characteristics of the data are pulled out, and the data set is mapped and interpreted as a whole. This is a systematic process whereby charts and research notes are reviewed, data (e.g. perceptions, accounts or experiences) are compared and contrasted, and patterns and connections are examined.
