# Supplementary material for: Smorgasbord or symphony? Assessing public health nutrition policies across 30 European countries using a novel framework
Source: BMC Public Health. 2014 Nov 21;14:1195. doi: 10.1186/1471-2458-14-1195 (PMC4251675; doi:10.1186/1471-2458-14-1195)
Supplement: Supplementary file 3 — Additional file 3: Qualitative Interview Findings. (PDF 1 MB) [file 12889_2014_7324_MOESM3_ESM.pdf]

Additional file 3
Qualitative Interview Findings

BELGIUM

Background

Public health in Belgium is the responsibility of the three communities (Flemish, French and German-speaking). Both the federal government and regional governments have ministers for public health. According to 2012 estimates, the average life expectancy is 79.65 years. Fat makes up 40% or more of the total energy in the food supply of Belgium. However, vegetable consumption is common, where 87% of women and 67% of men report consuming vegetables at least daily.

Findings

Most Effective Policy Options

Regulation, legislation, detaxation of healthy foods, and reformulation were perceived as the most effective policy options. Although respondents also felt that information and education campaigns were important for supporting change.

…legislation and regulation. Because with voluntary actions you will get little wins in the short term but not all actors will be moving and with regulation you get one rule for everyone so there is less inequity and yeah... Erm so the salt levels for example could maybe expand to other food categories, but that is what I would do. And then for example also minimum standards for the school meals, for the hospital meals. I think it's interesting to have also regulation for that. (Bel 2)

Information & education is still definitely the determinant of this for sure… In terms of taxation I would go for de-taxation of healthy foods - fruit and vegetables for instance. I think in some countries there have been the proposals to tax the fatty foods, is it Denmark? (Bel 3)

Huh it's difficult to select one because I think it's a combination of all of them. But the one which is most important I think is education; not education campaigns, education programmes in the food supply at school and at work… all that needs to be known by the kids and by the teenagers and also supported by a really good meal offer at school; and also at home of course but in the first instance at school or at work and like that you would have a continuous message….
Int: OK. So if you were the Minister and you had the power, what would you regulate?
Bel 4: (Pause) Er I would regulate probably the level of salt, sugar and trans fatty acid in the food.

Most Achievable Policy Options

The most achievable policy options were perceived as being reformulation and information and education campaigns. Both options were seen as being favoured by Government as they were relatively cheap and visible to the electorate.

Oh erm they will certainly support reformulation because it doesn't cost them anything. They will certainly have some information and education campaigns because that always helps for a good press conference. But I'm doubtful that they will have, for the reasons that I stated that if you regulate something which will impact on people's choice, I'm doubtful that that would happen, it might happen on some, for example limiting trans fats, that is something that people won't notice, but really for example increasing the price of fatty foods is something that is I'm guessing won't happen quickly. (Bel 1)

Reformulation again and what they always like is information and communication campaigns because they prefer to have visibility. That's also sometimes a problem because in the first place

they want some concrete visible action instead of structural changes but in fact it is always a combination of those options in fact. (Bel 2)

Current Challenges to Implementing Policies

Lack of competencies therefore leading to a lack of collaboration, lack of financial support and political will were perceived as current barriers to implementing polices. Together with an uncertain political situation and lack of cohesion between different stakeholders.

Yes indeed was in fact also part of the evaluation that it was fairly clear that we were dealing with a very difficult political situation so in some areas, for example especially in the area of communication and information, where we were dealing with, we were conducting a lot of information or communication campaigns to the general public and we developed food guides and food brochures, food leaflets but the communities didn't appreciate that at all. Especially the community of Flanders and in fact after the launch of those brochures they didn't want to collaborate anymore at the federal level… Because they saw it as their competency. Everything was to do with competencies. In fact according to them, all campaigns are prevention and should be dealt with by them. (Bel 2)

…So the point is that the federal government is still responsible for the very large majority of the finances that are related to healthcare, the communities are responsible for public health but the financial support that they receive for that is really minimal and not sufficient to develop really good national and community health programmes relating to nutrition… (Bel 3)

You have opposite partners so for instance if you take the group of breastfeeding issues and food of young infants, of course you have the side of the food industry and on the opposite side you have the breastfeeding associations, paediatricians and this is very unbalanced of course because they have different goals… .(Bel 4)

But for our part here in Belgium it was difficult because since 2006 we have a very uncertain political situation and it was quite complicated for one health minister to have a very strong position on a specific domain... it was difficult even to have media action because it was interpreted as a way to profile one minister or a way to make an event on one minister; but it wasn't the purpose - it was the purpose to make an event of the subject but it was complicated. So the French community for instance boycotted one of our actions on salt and in the Flemish part there were a lot of articles in the media and TV broadcasts, but in the French part that was interpreted as a political campaign. So you see, with regard to the media it was difficult really to have a strong presence, to be very visible was a problem for us during the last 5 years, due to tension and also due to that stupid difficult political situation… (Bel 4)

Effectiveness of regulatory vs voluntary approaches

Responses were mixed. One informant stated regulation is needed, one stated voluntary approaches are more effective as they are easier to implement and one stated both approaches are required to be effective.

No it might be effective (voluntary reformulation) but I'm not sure that it is being done in a substantial way. It's only being done for those food products where the company can use it as an additional unique selling point, to say that they have less fat content than the other company. So it

will work for a number of foods but it will not work for the vast majority of foods. Regulating the levels of fat and sugar in foods would be more effective, forcing food companies to do it (Bel 1)

Well you can do that for instance for the smoking ban and things like that, where you can control, but regulating the food intake of individuals is so difficult and also there I think some people will oppose that, not only because it is not important for public health but just for reasons of privacy and the government shouldn't lead too much in the choices of individuals. They can make a healthy choice, the food IS there, and that should not be regulated from the top, but done from the bottom with information campaigns and then potentially financial incentives that could be given to healthy food. Of course they could do it for instance in schools and also in occupational aspects, like the meals that are served in schools and big companies; there you could put some regulation and try to promote healthy foods and regulate for instance that in the schools that just outside of the schools the children are not able to buy coke and other soft drinks. So there regulation can I think be helpful to a certain extent, but not at the level of the individual I think. (Bel 3)

Er you can't regulate everything. So this is not regulatory on one hand and on the other voluntary; you need both again. (Bel 4)

CZECH REPUBLIC

Background

In the Czech republic there is no officially adopted policy document, which would solve nutritional policy of the state. There is a long term programme to improve the health status of the population of the Czech Republic - Health for All in the 21st Century (Health 21), adopted by Government resolution in 2002, where food is handled as a sub-task.
There are etablished dietary guidelines for the popultation public with rules that correspond to the existing knowledge of the science of human nutrition and meet nutritional guidleines for Europe as developed by WHO.
There are no incentives for healthy lifestyle (fat tax, junk food tax, tax for sweetened drinks etc).
There are nutritional standards for school meals and reference values for nutrient intake (DACH).

Findings

Most Effective Policy Options

Informants regarded regulation, legislation and taxation as the most effective policy options. Regulation in schools and the workplace was mentioned as a way to target specific groups. Information and education campaigns together with food labelling were also perceived as being effective for direct targeting of the population.

I think the regulation through the laws, through the legislation…I would legislate of course on the control of...first I have to have it labelled, I have to see what is in foods and I have obligatory...Obligatory, compulsory food labelling. At the moment I have labelling and people should be instructed what is good and what is not good and further legislative steps should be taken against unhealthy food and in this people are not very happy about the EU, but it is quite clear that without the EU things would be even worse. (Ch 1)

I think it is necessary to adopt more laws because the people don't want to eat (laughs) more healthier. Of course it may be one reason is also money because, or not law, maybe taxes on, higher taxes on junk food and therefore the healthier food would be cheaper. (Ch 3)

… I think er nutritional recommendations for school canteens and it is necessary to adopt in law I think. Not only recommendations but law for nutritional content of lunches. And to ban vending,

some content of vending machines in schools. Erm (pause) maybe (pause) so I would recommend that many companies also, so many companies use for children's food er signs or labelling like they write, "This food is suitable for children," and sometimes it is not suitable for children and we have no recommendation or no documents which is suitable for children. Companies can use it. (Ch 3)

...Er actually legislation and taxation go together. So er (pause) taxation that is sure because you have many positive things and you pay nothing. The taxation gives you some financial profit and it changes the customers' habits. The labelling is fantastic for informed people and they have to be educated first and once you get them educated they can make their own choices and so you are always respecting their liberty, always respecting their own choice but having them really informed. What we do until now is like to treat someone without letting him sign informed consent. (Ch 4)

Most Achievable Policy Options

Respondents felt that information and education campaigns would be the most achievable policy options. Legislation in relation to trans fat, salt and fruit and vegetables were seen as achievable, albeit harder to achieve.

I think that information and education campaigns because it's not really depending on the good will and financial possibilities and political will but rather on the good...I mean it's not depending on the good will of some elected people but mostly on the good will of some volunteers or teachers or health professionals and I think in the small sample size we can really have probably the most achievable target in the information and education campaigns. (Ch 4)

Current Challenges to Implementing Policies

Political will was perceived as the main barrier to implementing policies

Of course financing to support the prevention would be very reasonable and money-saving, but this is against the thinking of this Government. This is no conservative government; this is a liberal government that rules. And conservative people have some values and they are not against the poor people and so on; they have some sort of responsibility. But these do not have any responsibility for the public good. (Ch 2)

(__ __) and others opened up a question of regulating the market and putting through sensible health policies, and the Minister was basically not interested… he must know that it would be good to have it but I would say he is under political pressure….It is in the hands of the Finance Minister, who is leading the party and effectively rules the other ministers from this party and maybe from others as well in the Government; he is very strong this Minister of Finance and he is definitely the sort of man for getting money in his pocket and getting liberal policies; so this will not be a man pushing prevention…(Ch 4)

Effectiveness of regulatory vs voluntary approaches

Regulatory approaches were perceived as being more effective and cost effective than voluntary approaches.

ENGLAND

Background

England is part of the United Kingdom (England, Ireland, Scotland, Wales). The average life expectancy of people in England is 77.5 years for males and 81.7 years for females. Cardiovascular disease is the main cause of death in England. In 2007, cardiovascular disease led to nearly 159,000

deaths (accounting for nearly 34% of all deaths in England). This includes 74,185 deaths from coronary heart disease (CHD) and 43,539 from stroke. Despite recent improvements, death rates in the UK from CVD are relatively high compared with other developed countries (only Ireland and Finland have higher rates).

Findings

Most Effective Policy Options

Legislation, reformulation and taxation were perceived as the most effective policy options. One respondent also commented upon information and education campaigns and labelling being effective for directly informing the population.

Well I suppose the answer to that is they will be the policy options which operate at the population level, and that's what the evidence supports. And that points to reformulation of processed foods, price, availability, marketing. So the sorts of things, and food standards. So the standards for school food for example, standards for public procurement of food in public institutions …(Eng 1)

Oh probably some kind of tax and I would no way know the detail of all that but if you could price unhealthier food higher, I think that particularly currently more so, and more so in the future again, the price of food is incredibly important so people always whinge, sometimes rightly, sometimes wrongly that healthier food costs more so it should be the other way round; unhealthy food should cost more, if we could any way achieve that I think that would be the most powerful. (Eng 2)

it's about trying to tackle whole populations rather than individual approaches. So just by the nature of that, because that's what I think is the right approach, because there's an inequality issue and all that sort of stuff, and I suppose I'm more pro-regulation legislation and reformulation and stuff that gets everybody rather than asking people to make individual choices. It's about saying OK of course people have the right to information, and obviously quite a lot of the work that I've done has been around things like food labelling; I think if you're going to provide people with that sort of information it should be understandable and usable and appropriate. You have to do other stuff that means that people can make those choices in a context where they're able to make a healthy choice if they want to. I think they should also implement traffic light labelling probably. So those would be effective in some way about helping to change the landscape a bit. (Eng 4)

Taxation of foods in relationship to their health and we could reform VAT and that wouldn't be too politically unacceptable. We could have a soft drink tax as they're proposing in France. I think it is possible to make certain changes to certain areas. I don't think these changes are gonna happen in the next few years but possibly thereafter, midterm of this Government. (Eng 5)

…but I think that kind of information basically empowering people to make healthier choices, well not even to make healthier choices but to understand the choices that they are making in the first place and then if they choose to make an unhealthy choice at least it's an informed choice. I think that's one of the most effective ways. Rather than, because I think generally on the whole people are aware of what is and isn't healthy to an extent. I mean there's obviously some things that they continue to be surprised by like household cheap bread but generally people know that fruit and veg is good for you and kind of a greasy fry-up's not so good. But it's about kind of how can they understand how to eat a balanced diet and if you have some unhealthy food what could you have to counteract it and is everything you're eating healthy? So I think labelling is really important. (Eng 6)

Most Achievable Policy Options

Banning trans fats and food labelling (additional to the EU scheme) were perceived as the most achievable policy options. Other achievable policy options included pre-school nutrition and controlling food advertising.

I suspect the pre-school nutrition might be actually, if we worked on that a bit because we've gone a long way towards that And there is more awareness of it and the very fact we've achieved it in school; I'm not saying school's perfect but it's a lot better, but I suspect we could possibly achieve that in pre-school. I also can't really see why trans fats can't be banned either. I mean I think that's a political choice more than anything else. Also labelling, it is to a certain extent the European Union function, but this Government could still have its own scheme so it could be done. So I'm aware there's national and European confidence too, but anyway.
(Eng 2)

Stronger control over marketing of unhealthy foods to children, I would certainly put that high as a priority. It's controllable, it's achievable so when you ask me about most achievable, I'd actually possibly put that up there as perhaps the most achievable. It clearly can be done through control over advertising. So that should be there. (Eng 3)

Yeah I think some of the lessons on salt could be applied here and also labelling would potentially help as well. And possibly restrictions on marketing could help. So things around labelling and marketing and secondary to those would be reformulation…. I think they're achievable but I'm not sure there is the political appetite. I don't think there's any insurmountable barriers to them but I'm not sure there is political appetite for them at the moment and obviously there is the economic side of things in terms of policy context and there is an understandable reluctance to do anything which could be perceived as anti-business, anti-growth. (Eng 6)

Current Challenges to Implementing Policies

Responses regarding barriers to implementing policies were mixed. Perceived barriers included, lack of political will, support, consensus, resources and research evidence. Together with the food industry having too much influence over political decisions.

I suppose it's about resources, it's about political will, it's about whether or not there's consensus, even in your own community, whether there are territorial differences; I don't know.(Eng 1)

…Government at the moment is that I would argue that they're working far too closely with food producers. I'm not at all against encouraging food producers to change what they do and we're seeing some quite positive changes, but I think they're addressing a market need more than actually being pushed to do things because of health.

…it was quite shocking the lack of knowledge and understanding of how the CAP could impact on health I have to say… So I still feel, and because of the focus upon obesity as opposed to healthy eating, there is a block because professionals don't always see food or poor diet as a bigger risk factor as it is for NCD. When you see fast food companies paying the kind of money they are to be associated with the Olympics, you can see how important it is for them to have that kind of image. So you know, the food industry will fight tooth and nail for any kind of regulation really…(Eng 2)

So you know there's a broad issue in food about how do you get the industry to work in support of public health goals and the challenge in doing that is a barrier for pretty well all the food policy work you want to do. However at the level of individual policies, the barriers are all going to be different. In salt there is a toxicology issue, there's a safety issue, there's a consumer palatability issue, there's in some cases a technical challenge in reducing salt. Those are all barriers but when you're talking about trans fats then actually for the vast majority of products those are not issues, it's a matter of cost. That would be the biggest barrier to that. And it's about effective audit strategies and it's

about reach; how do you get to the kebab fan? So the barriers for each policy are going to be different. (Eng 3)

You know there's almost no food policy frankly, let alone evaluation of that or implementation of information and once you've got that, evaluation that's sensible and not rubbish. Because quite a lot of it's incredibly poor quality when you get to read them. What's the point of doing that evaluation anyway? And actually quite often that evaluation doesn't include any element of cost effectiveness at all….So you can understand why things don't get funded but it's incredibly frustrating because then of course people always say, "Well what's effective then?" Well if you're not gonna fund the studies that tell you that, then how can you tell policy makers what's effective or not? And you just end up getting back to asking the same experts what they think.
(Eng 4)

I think there is issues around the consensus of what needs to be done and it's an interesting one because on the one hand there's a lot of anxiety from all policy makers and all politicians of all parties, certainly in England, anxiety about rising levels of obesity but then there isn't a consequential urgency and you know there's been various obesity strategies and public health documents, but there hasn't been anything really, really (sighs) I don't know, really, really radical I guess…(Eng 6)

Effectiveness of regulatory vs voluntary approaches
Regulatory approaches were perceived as being more effective than voluntary approaches although voluntary approaches were perceived as being easier to achieve.

I don't think legislation is necessarily more effective than voluntary action. I don't think education is less effective than changing the price of foods. I think if you're thinking that, I know some people who think that, and coming from where I am coming from you might expect me to think that legislation's more effective than voluntary restriction or that price changes are more effective than taxation, or more effective than education. I just don't know. I think it's almost impossible to say to be honest. But I think the distinction here, the 2 axes that you might think about here is the voluntary versus mandatory and things which do stuff about or try to change, get people to change through giving them information and some change which involves trying to change the environment in which people are making choices. (Eng 5)

I think they're (the government) unlikely to go for legislation or taxation. I think they're more likely to be interested in kind of voluntary activity from industry and I think possibly kind of influencing and action at a local level is perhaps more likely to be happening rather than a centralised approach. (Eng 6)

ESTONIA

Cardiovascular diseases (CVD) are the main reasons for early loss of work capacity and death (at age <65) in Estonia. Estonia is leading in CVD caused deaths in Europe. Each year, at least 250 men and 80 women of every 100,000 die of CVD before the age of 65. The average life expectancy in Estonia is 73.2, while the European Union average is 78. The rate of deaths from diseases of the circulatory organs in Estonia is 3.5 times higher than that of 'old' Europe, and the number of deaths caused by external factors is 4 times higher.

Most Effective Policy Options
Legislation, regulation, taxation and subsidies were identified as the best policy options. With food labelling and information/education campaigns also being valuable.

Legislation/Subsides and Information/education campaigns - vegetables should be subsidized they are too expensive. Estonia needs nutrition curriculum, we do not have possibility to study nutrition in Estonia (Est 1)

Through legislation, regulations and subsidies to make healthy food more available and accessible for people (Est 2)

OK it is somehow difficult to…I mean if there is taxation or subsidies, there should be legislation and regulation, so you can't divide them. There is also one thing with regulation and legislation, like for example for school fruit scheme we need legislation as well. But I think that subsidies might be one possibility just to offer cheaper healthy foods. Or to subsidise to influence farmers to grow fruit. .. (Est 3)

It's very hard to evaluate for me but legislation is important and regulation regarding children's nutrition habits, and labelling also is important. And of course information & education campaigns are good. (Est 4)

Most Achievable Policy Options

All informants regarded information/education campaigns as being the most achievable policy options, but regulation, taxation and subsides were regarded as being the most effective and cost effective.

Information/education campaigns (Est 1)

It depends from political will and priorities of the government. If there would be political will – all these policy options would be achievable (Est 2)

I think we need to continue with education so if the population is informed it is a prerequisite that they do the healthy choice. So continue with nutrition topics in terms of pregnancy and the whole life cycle. (Est 3)

Current Challenges to Implementing Policies

Political will, lack financial and human resources, and lack of collaboration were regarded as being the main barriers to developing and implementing policies.

Political will. Our country is lead by the ultraliberal goverment their opinion is that health is individual responsibility – the government will not intervene... (Est 2)

…it depends on possibilities, on money, on willingness and how sub actions are implemented. I think the barriers, the biggest barrier is lack of competent personnel in the area of food and nutrition. We have key curricula of food technology and nursing, teaching, but we really do not have curricular health & nutrition and there are very limited number of experts working in this field. And that makes it also difficult to have good analytical papers, have good campaigns, have opinion leaders, spokesmen and so on. (Est 3)

I just thought about it and I think the problem might be if there are several policies with the same aims, like in Estonia we had this; that's why ministries should be more integrated as a whole and I

think the collaboration between the Government and these organisations and the private sector is very important. (Est 4)

Effectiveness of regulatory vs voluntary approaches

All respondents perceived regulatory approaches to be more effective than voluntary approaches.

Yes I think because the developments in national health in recent years have been positive. Life expectancy has increased more rapidly than the other European countries. And the progress over the last decade has also seen the national healthcare system become one of the most cost effective on the continent. (Est 1)

They might be because Estonia has a history of regulatory measures from the Soviet Union and some are willing to have legislation and regulation and after that voluntary actions, for example by the food industry or by the restaurants. It depends on how informed the population are. If they are willing to have the healthier products then they would do those kinds of things but that's why for example for reformulation and subsidies, I think the regulatory is better. (Est 2)

FINLAND

Background

Dietary changes in Finland during the last 30-40 years have been pronounced (less fat, saturated fat, salt, 3-4 times more fruit and vegetables). The most recent FinDIET data (2007) show mean serum cholesterol levels have decreased by 20% since 1975 with changes in dietary fat composition explaining 60-65% of the decline. The 1982-1997 age-adjusted CHD mortality declined by 63%. 37% of this decline was estimated to result from serum cholesterol lowering.

Findings

Most Effective Policy Options

Taxation, reformulation, labelling, information and education campaigns and subsides were deemed the most effective policy options.

Erm I think we need all of those because we need to have a package of different tools and they are different kinds of tools, which all have their own place. So I don't know that any one should not be included. I think sometimes it's more; it's too much focus on this information and educational campaigns. We need those too but these don't work alone, so we need these kind of structural solutions.

T's hard to say, it's hard to say which one. I think we need legislation and regulations because those working within the food industry need some carrots as well as sticks; that's important. Reformulation is really important too. We need products which have better fat contents and this applies especially for salt content; it's really important. And labelling is too important for some people but I think this is not the solution for all. We need the information but we have many people who don't read the labels and that's why we need the labelling on the front of packages and the symbols are important. And of course information and education campaigns are also important. (Fin 1)

To tax the unhealthy products and subsidise for example vegetables because people are really looking at the prices when they buy food. Reformulation has gone a long way already in Finland. I don't know how would they, because we have a lot of low fat, low salt products. What else could we do? I don't know. Labelling comes from the EU (laughs) and we can't do very much about it.

Information and education campaigns, campaigns are very good but they are so short. You have a campaign for 2 months or 1 year and then it stops and then nobody remembers after that. (Fin 2)

What should ideally be done?" I think we should do something that is combined with previous activities and I think guiding catering in day care, schools and work sites is a very good area and we could really do something. And you have heard about this catering guiding system? And in schools I think this assessment of the school lunch is indeed something that should be done above regulation. It should be part of how you, I don't know what they call those teaching programmes, education policy? It should be something combined with that. (Fin 3)

I do think that in some areas we should get more regulation here again, with salt and also I hope that we could move in the taxation issue with saturated fat, salt and sugar. (Fin 4)

I think the taxation is very effective. I would go to taxation of the sugar because it is how it is used now and how people drink the soft drinks and eat candies so I would tax it more. And I would subsidise the food from Finland, but it is not possible in the EU (laughs) so these subsidies we have to agree with the EU. I would go for information and education campaigns because those are maybe in the long run the most effective ways. (Fin 5)

Most Achievable Policy Options
Legislation was perceived as being difficult to achieve. However, taxation was perceived as being achievable together with information and education and better food labelling.

Erm from our experience of the formulation of this heart symbol system we have administrated since 2000, I think reformulation is, erm we have had good progress in reformulation, that's one. Er legislation during these days is not that easy anymore because we have this EU legislation and I will have to say this beforehand but erm that has been one barrier for Finland because we have been ahead of this food policy and nutrition policies and an example is the salt legislation. We had a good national salt legislation concerning carrots for industry, they could label the foods with the sign of less salt or erm I don't remember what was the correct word they could use, but because of EU legislation we had to get rid of that. (Fin 1)

Taxation would be achievable, which they are now thinking of. Because it brings money and all the taxation systems are not for the health, they are for the money. Which isn't a nice thing but...(Fin 2)

…I think actually information and education campaigns and labelling would be the most achievable. Obviously regarding labelling we always have to remember the EU legislation (laughs.)… But for example this heart symbol is national and the EU is aware of that and we have permission to use it so, and the European Food Safety Authority is currently working on the health claims and I think that work also will help the consumers and it's part of the labelling issue.…Legislation is so, it's like a tanker at the moment, it's very difficult to turn because of this EU legislation system. It's very hard to affect that and it takes time. It takes a lot of time so it's not very flexible at the moment. (Fin 6)

Current Challenges to Implementing Policies
Barriers to implementing policies were many and varied. They included the power of the media, including social media, European legislation replacing already effective national legislation, the influence of food companies and a lack of collaboration.

Mmm er I think one, I don't know if it's a barrier, maybe you could call it a barrier, it's that there are so many stakeholders and we do not have any…So sometimes when we're talking about healthy heart policies, other ministries are more powerful because they have the money. So it's not that

powerful… And one thing is that in Finland that most decisions are made at local level and that's the most important challenge at the moment. At the Government level there might be good co-operation with different ministries and different policies, but when we go to local level, so when we promote the idea of healthy policies, they might not see that in that way at all… (Fin 1)

I think indeed the unofficial activity of the social media and the media in general is something that is making the nutrition education so very difficult at the moment, in Finland. Yes and food marketing also because I think a good example is energy drinks. They have started some weeks ago in Finland an advertisement campaign concerning energy drinks for 7 year old children. The Finnish recommendations I mentioned here, it is recommending that children under 15 should not drink energy drinks. But then those food enterprises, they are starting production that is focussed on children who are 7 years old and so they are making somehow an illustration that even children need energy drinks and when they get familiar drinks with energy drinks at that age, they will continue and continue and continue. And this is only one example…. (Fin 3)

But the problem is that when you have to move to specific legislation or taxation, then the competing interest comes across strongly. So well let's take this candy and soft drink taxation. Of course the industry concerned was lobbying very heavily against that and fortunately they could not overcome so we got the tax; it's still fairly small so we hope to increase...actually the Government is increasing the tax, which is good. But then when we go to any specific legislation so there are all kinds of barriers, much of that is economic companies and it's the same kind of thing that you are discussing in the UK. Industry is always against regulation or new regulations. They are pretty powerful to the civil servants and specific legislation is not so easy. There is inertia and change and all kinds of problems for the civil servants. We find out that whenever we get the new legislation to the Parliament, the Parliament is much more pro-health because they listen to people, but the civil servants are in the middle of all kinds of constraints. So that is where the problem is. (Fin 4)

…one of the problems with the nutrition policy that it is so much linked to the Common Agricultural Policy of the EU so that is an additional issue that we have to follow EU policies. …We had just persuaded the Finnish schools to serve mainly skimmed milk and low fat cheese and the EU came and said, "Hey schools, you will get money if you serve fatty milk and fatty cheese." And so the Finnish members of the European Parliament were lobbying for several years before it was changed and of course the EU tries to have various kinds of things so that the excess fat is eaten by people. (Fin 4)

In Finland currently I would say there is this stupid discussion about nutrition. It involves so many people who are not experts and do not know the field and just have their own opinions and the media is considering them as experts so in Finland we really have a big problem currently. (Fin 6)

Effectiveness of regulatory vs voluntary approaches

Views upon the effective of regulatory versus voluntary approaches were mixed.The majority of informants believed regulatory approaches were more effective than voluntary approaches. However, it depended upon the issue (i.e. some issues benefit from regulation, for example labelling of unhealthy food as the food industry would never agree to implementing it voluntarily). Regulation was seen as being more effective by another informant, but may not be welcomed by the general population.

It depends. When we are talking about regulations in terms of for example labelling something which is not that healthy, so then we need mandatory regulations because industry wouldn't like to label or give the information if it's not that good for them. But those erm both of these things could

be voluntary. For example this heart symbol system that we have is voluntary and I think it works. So that's, it depends on the issue (Fin 1)

Erm I think on EU level the regulation of consumer information on labelling on food items and food control and erm all those things, they are erm they are making the environment of food production and giving regulation, how to organise that. And I have nothing against it. I think that is a good way to organise it and then I think what comes to the promotion of healthy food items, the voluntary efforts of the food industry are the best way. It is a tool for competition… But then I think this regulatory approach is needed in the good tradition of Finnish catering, like daycare and schools and work site restaurants and I think we are in a situation that we should evaluate those regulations needed. Last week it was in the biggest national newspaper an article on children's meals in daycare and in schools. The article was asking if it is as easy to get a vegetarian meal in daycare and schools in all areas of Finland and the answer was that it is not equal in some areas...(Fin 3)

It is a question of regulation and voluntary activities and what is the balance? The balance depends very much on what the people are, how much the people are ready, how strong the argument is, how can you persuade the politicians? And so of course I feel that we need, I would like to see much more regulation, much more, but it's not so easy. (Fin 4)

Regulatory approaches could work in, as I said in public catering services, in lunches and school meals, hospital meals and I think that has to be, there has to be regulation and information to the service providers. In salt the regulation started to work because it put the bakeries into a different position and labelling is very powerful and I think you have to go through regulatory means to start labelling foods. That is very powerful. (Fin 5)

GERMANY

Background

The German National Nutrition Survey II (NVS II) a representative survey on the nutrient and energy intake of 14–80-year-olds determined that one in five Germans is obese (BMI ≥ 30). Analyses on current food consumption, lifestyle and eating behaviour demonstrated that 36% men and 31% women exceeded the guidelines for daily energy intake for median physical activity. Of even more concern are the results for the daily fat intake: 80% men and 76% women exceed the daily fat intake recommendations (30% of total energy intake) (2008).

Findings

Most Effective Policy Options

Legislation, regulation and food labelling were identified as the best policy options within Germany by all key informants:

I think from the point of view of legislation and regulation, we would need some initiatives to have a change because we rely on companies and trade to do something voluntarily and this has not been effective in the past 10 or 20 or 30 years... (G1)

Well there has been great discussion on this traffic light thing, to put the red or yellow or green marks on food items, specifically salt or fat or sugar but this has been blocked at present and is not pursued.... (G2)

Taxation was regarded as a negative option. This was due to uncertainty about consumers' reaction and the current financial crisis:

To obtain a tax for example is difficult nowadays in Germany. People are very critical about paying more because of the financial crisis they feel they have to pay for the whole of Europe, and politicians won´t win the elections next year with the political target to rise taxes on food. (G4)

Most Achievable Policy Options

Two informants regarded education and information campaigns as the most achievable policy options, although not necessarily the most effective.

Certainly the Government would be most willing to do, would be giving some money for education and information campaigns, which in my opinion would be the least effective (G1)

Forcing the food industry via legislation and regulation to reformulate food was thought to be achievable and one of the cheapest ways for government to achieve improvements in health. However, informants were uncertain if political will existed to enforce such measures.

… legislation, reformulation and therefore forcing the industry to change their products would be one of the possible solutions.(G3)

Right. And how feasible do you think that would be to achieve?(interviewer)

Er no idea. it would be possibly the cheapest way for the government to change something, how feasible this is depends strongly on their will/commitment to do it (G3)

Current Challenges to Implementing Policies

All key informants felt that political will was the main barrier to developing and implementing policies:

I think there is a lack of political will or let's say there are always more important issues. So in the political parties, to get organised everything else, but food and prevention has a low priority (G2)

I think first Governmental attitude, namely the promotion of self-regulatory approach, especially the Government at this time, especially the party FDP; they are very, very erm keen on having self-regulatory approaches. They want that everyone is responsible for himself and they don't want any regulations made by Government...(G3)

There is a lack of political will; I think this is really crucial and in Germany the prevention strategies are mostly based on individual measures...(G4)

Effectiveness of regulatory vs voluntary approaches

All respondents perceived regulation to be more effective that voluntary measures. One respondent stated that voluntary actions have been in place for over 30 years with limited effect. However, resistance to regulation was perceived as being strong:

Yeah but erm there's also a great...I mean there are great restrictions for regulating diet and I think the resistance would be strong in many parties and I don't see that real regulation...maybe aside from the trans fatty acid issue...will be coming. I don't think so.(G1)

Definitely, yes. I think so. The ideology of freedom of choice is highly appreciated here which of course is good but I think it merely is the freedom to get ill today (laughs). It is the freedom to having the choice between different bad opportunities, that's often the reality. People don't see that

prevention is also about taking away some of the bad options and enabling more freedom to healthier options. (G2)

GREECE

Background

The dietary habits of the Greek population have changed considerably throughout the past years. There has been an increasing trend towards the adoption of more "western type" diets with the majority of the population failing to meet the Greek nutrition guidelines. The gradual decline of the traditional Greek Mediterranean diet - which is globally accepted as one of the healthiest diets – combined with the significant reduction of physical activity, have resulted in increasing obesity levels and other nutrition-related disorders. Greece is reported to have one of the highest prevalences in childhood obesity with significant rising trends.

Findings

Most Effective Policy Options

Greek Key Informants varied in their perceptions of the best policy options for their country. Three felt that Education and Information campaigns were key, although two acknowledged that this should be supported by legislation, taxation reformulation and/or food labelling reform:

I think legislation as you mentioned there, when it comes to food advertisement to kids or promotion of food... there should be some regulation for the food allowed in schools. There should be a supportive environment, like facilities for food preparation in schools.... The other issue is also taxation. I think that the taxes on healthy foods like fruits and vegetables should be lower and taxation should be higher on unhealthy snacks. (Gr1)

I think the most important is information and education campaigns. The second could be reformulation of the products. Now labelling is something that is evolving because the regulation on labelling has changed so the information on the label will be much more clear in the next 2 years. And if all these don't work, I think the next step is legislation and taxation. (Gr3)

Of course we have to improve for example something in relation to information and education of the public in relation to healthy diet. Then I'm sure that some taxation issues and legislation issues...Of course, of course we need, we definitely need legislation because I think it will very much help to implement a lot of our policies. Maybe some issues related to the price of the food so some taxation issues also would be important to look at more closely (Gr 4)

One informant commented that legislation administered at the European level was sufficient and translated into National policy:

We are OK with the European Union, we are obliged to transpose European legislation in our national legislation. We don't need anything more about that. (Gr2)

Most Achievable Policy Options

Although difficult to achieve, informants felt that targeted legislation, regulation and taxation would have the greatest health impact and should be pursued:

...a very easy thing to do, would be if you isolate toys from food but I feel there would be a lot of complaints by the food industry on this issue. The most effective tools in most cases are the most difficult I would say. ...legislation for schools and school programmes for health promotion and

catering in schools and facilities in schools like a freezer, and to devote a certain time, you know one hour per day for a healthy lunch, could be achievable I would say. (Gr3)

Legislation, maybe it's not so much achievable for example information and education campaigns are more achievable. Legislation is sometimes not very easy to do, although it would be very effective. (Gr 4)

Current Challenges to Implementing Policies

Political will was identified as a key barrier to developing and implementing policies together with a lack of scientific evidence, and lack of time and resources:

There is no political will. They have no interest in people's health. What they care about is their personal promotion and their personal presentation in the mass media and being seen to be doing something, but they never outline a serious policy. They never have some indexes to estimate the results… my general aspect is that my Government have no official health or nutrition policy. (Gr 2)

The only thing is that the people, the industry are unwilling to implement the policies that we develop. It's very difficult to get the scientific data to prove that it is good to develop the policy. This is the most important barrier. (Gr 3)

I have, from my experience, because I have been involved in one policy… I think there were problems in there was a committee that developed the policy but there were, I could say there was lack of time to be prepared for, to prepare the policy, lack of time and resources, both in relation to expertise. There were few people who had the background and the knowledge to contribute and also because these committees were set up by themselves and we had to work you know extra time to develop this policy, I mean besides our work…. There were not a lot of people from the Ministry that could help us. So I think that was the main barrier for me. (Gr 4)

Effectiveness of regulatory vs voluntary approaches

Respondents were mixed in their views of the effectiveness of regulatory versus voluntary approaches. Two respondents felt that voluntary approaches were not effective and regulation was needed. One respondent commented that both approaches were required and one respondent felt that regulatory approaches are effective for children and schools, but not everything can be regulated and probably not effective for industry.

Well I don't believe in voluntary approaches. I have a bitter experience, not only in the food sector, but several sectors. We are involved in a long time with no results and my feeling, or better OUR feeling, and other consumer organisations in Europe, we have the same feeling and they have found to not implement their own decisions. So if we need something, some regulation, I think this should be er obligatory. (Gr 1)

I think this is tricky because erm I think for some issues that we believe that there is a population that is let's say sensitive, such as children, and maybe infants and some people that are in particular stages of their lives, there we could use regulatory approaches like the one that I told you that we used at the schools for example. There we have a regulatory approach and we don't permit some foods to be sold there. But because we are in a society that is a free society, you cannot regulate everything. It is important to...I'm not sure that the regulatory approach would be effective for the industry for example. I'm not sure we could use it there because it's the free market. (Gr 2)

Because erm (pause) if you leave it voluntarily, what I have seen from my experience before is the most educated parents and the most wealthy in society, the upper economic groups, they are more sensitive to health promotion, to health issues. If you leave it voluntarily, they certainly will adopt it. So again you need, that's what I am saying. And I think our record should be to minimise social inequality and health inequality. So we need to ensure that we start with the most vulnerable in society. (GR 4)

ICELAND

Background

Since the 1970s there has been a sharp decline in whole milk, butter, lamb, mutton and fish consumption, mainly replaced by chicken, pork, vegetable oils as well as increased F&V consumption. Since the collapse of the banks in October 2008, Icelanders have lower purchasing power and face increased prices. From October 2008 to October 2009, vegetable prices rose by 45%. Other commodities rose less. (Steingrimsdottir press realease, 29.04. 11 'Changes in the food habits of Icelanders' on the PHI website).

Findings

Most Effective Policy Options

Legislation, regulation, taxation, subsides and Information and education campaigns were perceived as the most effective policy options.

Well starting from the bottom of the list, I think we could strike out education and information campaigns. OK they are good but they are not going to be very effective. So I think the most effective points would be legislation, regulation and taxation. Top of the list. And of course subsidies, that's politically...it comes and goes…So that's always, those things are always changing but taxation of course and regulation yes. (Ice 1)

I think legislation and of course good information to consumers and I think it's very important. It's education in the schools, how to live a better life, how to eat to be healthier and so on. It's very much discussed here in Iceland that young people are eating something that they shouldn't eat so much like fast food for example, and they are sitting when they come home from school and they sit down in front of the computer and they are sitting there and they should have more motion. And the young people in Iceland, it is more and more a problem that the young people here in Iceland are too fat and I think one of the reasons is the food they are eating. And also that they don't exercise. So I think we need more education in schools about this, how important it is to choose the right food and to exercise. So we call on that in schools. But also it's important information for older people too so we need information and education not just for children, but also adults. (Ice 2)

To tell you the truth I couldn't tell. There has been a lot of discussion about this so taxation of course we know that if you have to pay more for the food you are less likely to buy it so in a way that will surely work and actually it works for smoking. We know that. Subsidies, subsiding something is another thing. If you pay for...if the healthy food gets cheaper yeah but then you have to fight also the industry, so why are you reducing my success of surviving as a company by giving that other company something? So there is a lot of tricky things in there… And then information and education campaigns. Well I know why but I think continuous information and lobbyism rather than campaigns about let's eat more fruit now. But it delivers. It seeps into the minds of the people and they may not eat 5 fruits a day, but they may eat 3 rather than 1. (Ice 3)

Well it is of course like legislation and regulation on trans fatty acids; of course it has an effect. And you don't have to educate people or anything. (Ice 4)

… If there is the legislation to have information on nutrition on all packages and if it's legislation to label some kind of health labelling, you know, all different kinds of stuff; I think they take it very seriously. It's important but it has to be a majority understanding for it. Otherwise it will become unpopular. So honestly I cannot decide from my point of view right now because my point of view is only the thing that I would try to find out, which would be the most important for the population; which would be the most powerful. I think all can be used. (Ice 5)

Most Achievable Policy Options
Legislation, regulation and information and education campaigns were perceived as being the most achievable policy options.

We use a lot of sugar here in Iceland. We are consuming a lot of sugar. I think it's very important to try to influence consumers to use less sugar than they do today. (Ice 2)

I think its more information to consumers. We call for information number one, how to live healthier lives when you use less sugar and salt. I don't believe in taxation. I think information is more important than taxation on sugar. Maybe I'm wrong but the organisation is against more taxation on food because food prices here in Iceland are high enough. So we talk more about good information, good campaigns against the use of too much sugar and salt. And it has started already in schools to tell the children how unhealthy it is to use too much sugar and salt. I think we've got more out of it with information already in schools than a little bit of a higher price. The people would buy it anyway. I think it would have much more effect to have good campaigns and good information. I think it's the best way. (Ice 3)

Salt is not difficult to improve on if you just put up some regulations (Ice 5)

Current Challenges to Implementing Policies
The influence of industry was perceived as being the key barrier to implementing policy. With political will being a strong second challenge:

Erm very unstable politics I guess. We have Government that has been ruling since the crunch in 2008, I think from 2009. They are er they are very occupied of course with the economic situation or the economic issues…and they didn't care; they didn't...you know you couldn't get access. Others were very accessible, you could get an appointment and we sat down and talked about things like tobacco and cholesterol and stuff and policy based prevention, but then before you knew it they were out of office and then a new guy came in and you had to start all over… (Ice 1)

Sometimes industry is stronger at...they have more money to lobby against what we are saying so that's maybe the biggest barrier when they don't agree with us. (Ice 2)

I might guess that it was part industry and lobbyism with politicians (against a sugar tax) so lobbyism is crucial. We look at it as useful when it is us who are lobbying for better lives and health of the nation, but the other people have alternative motives, although they will never admit that and actually if you talk with the individuals, they are equally concerned as you and me but leaving their brain on the shelf when they go to work. So these guys lobby also for their own interests. (Ice 3)

I think the old difficulties, which may be changing now, I'm not sure though, are those related to that we have to do what is the best for industry. This is actually the old view I think, that you have a kind of er local industry, or industries in each country which are probably strong and companies maybe have been important in giving taxes and finances to the official finances of the Government (Ice 5)

Effectiveness of regulatory vs voluntary approaches

Legislation/regulation, taxation and subsides were perceived as the most effective, with some respondents perceiving information and education campaigns as also being important to support regulatory changes.

Because these things, the freedom of choice isn't all that free. The choices, you know the default options are made for us everywhere. We are not aware of it but the decisions are already made for us and I think we have to...especially regarding the kids and there is nothing in the Icelandic law that says that you are not allowed to advertise in the media junk food to kids. (Ice 1)

We have an agreement that is like you were saying non-regulatory, or self regulation, and they say "We will, we will do that" but they don't do it. So we have many examples of those things, about marketing to children and they have said they are not going to market to children and still they do it. So we don't trust industry on that…Yeah so we say it just has to be in the laws or in the regulations. (Ice 2)

The change in dietary habits all over the world are because of lobbyism or information to the people and to believe that doesn't work is not true. We have evidence. We have evidence for dietary habits but now we are faced with different sorts of problems with this increased obesity and the consequences of that, which is diabetes. And I think we should not underestimate the importance of obtaining information and relaying the information to the people. It may not be enough but it surely is going to be the core of what's being done because you can put all the regulations and rules you like, and if the people don't like those rules, they don't follow them. So rules are meaningless unless you do something to accompany them with respect to informing the public. (Ice 3)

Erm (pause) of course you can get the effect right way if you use the legislation (sighs) but er well I'll just think about the relation between the Government and the industry (pause) so this er (pause) it would be ideal to keep the relation good and have the voluntary way and...but of course if the industry doesn't do those things, then it would be necessary to use legislation. (Ice 4)

IRELAND

Background

Cardiovascular disease remains the most common cause of death in Ireland, currently accounting for one-third of all deaths and one in five premature deaths. However, there has been substantial progress. Age-standardised death rates from cardiovascular disease have decreased by two-thirds over the past 30 years. Despite improvements, Ireland still ranks below the EU15 average for life expectancy for both men and women. As mortality rates have reduced, demand on health services has intensified.

Findings

Most Effective Policy Options

Taxation, followed by reformulation and legislation were perceived as the most effective policy options.

I would, in terms of powerful options, I'd say I'd probably go for reformulation with the whole fiscal measures as being kind of a close second behind. Not giving an equivocal answer! I think because reformulation can be so broad reaching but in saying that there's obviously technical challenges but in terms of the potential power I would think I would go for reformulation. (Ire 1)

Well I think we need to use all of those tools (legislation/regulation, taxation etc); it's which are the highest priority. Obviously there has been a good bit of work done on the food labelling issue and we are to some extent hamstrung by the EU consensus and by what I regard as the absurd guideline daily amount regime that we are subjected to. And I think that we need to continue to lobby for a traffic light system. (Ire 2)

It's a balance. It's like tobacco. There's no silver bullet. There's no one answer; it's gonna take combinations of them all. Where appropriate like we have been pushing for the tax rises on soft drinks and I suppose we all got undermined with the change in policy in Denmark. In fact it wasn't a change in policy at all when you looked at it, they had also put taxes on 5 a day products and then withdrew them because they got the model wrong, but they actually didn't remove the taxing function on sugary drinks, although we all have the perception that they did. So there's issues like that, so taxation and some of those issues should matter… (Ire 3)

Well we've very little legislation around food. We have voluntary calories on menus; taxation we really have none - in fact our current taxation system is already an anomaly because under our current VAT we have a medium tax or a mid tax. Our full tax is around 20% or 12% and we actually have a lesser tax for products like croissants and banoffee and some amazingly high sugar and high fat foods. So our taxation is an anomaly and yet the Minister of Finance feels that because there's VAT on many of the foods, junk foods we're talking about, that he feels it's not necessary. He's missing the point. We need a health related levy. (Ire 4)

Most Achievable Policy Options
Taxation on sugary drinks and other products containing sugar and salt were perceived as being most achievable. Information and education campaigns for increasing fruit and vegetable consumption were also mentioned.

In terms of achievable I would go with the fiscal measure and the sugar tax because that seems to be quite likely to be actually something seriously proposed by the Minister in the next, the budget is coming up in a month's time and I understand it's going to be proposed as part of that. (Ire 1)
No I think in terms of developing national policies, I think that salt is still the most feasible and practical because we can do a lot directly, you know we can say to industry we are putting a cap on the amount of salt that you can add to bread and cereal and so on. We can through public sector purchasing both in the health sector and in local government etc. (Ire 2)
Yeah I think you probably could get taxation on sugary drinks, and that would be hard but I think we could…Yeah I think information campaigns or whatever on improving consumption of fruit & veg is practical and feasible. I do think the argument around sugar is coming our way. I think it's moving in the direction that I think you could make some inroads there.(Ire 3)

I think the sugar ultimately because sugar is no nutrient and I think the evidence is building about the evidence around high consumption of sugar and then sugar linked with obesity, weight and as I say empty calories. So I think sugar is one, and fruit & veg. (Ire 4)

Current Challenges to Implementing Policies
Views upon current challenges varied. Informants commented upon a lack of a National Nutritional policy since 2004; a lack of resources, leadership and intersectoral working; a lack of expertise and public health capacity and the power of the food industry.

I would think resources, which is a very important issue at the moment needless to say, and even back in the boom times it still was an issue to some extent, but very much so now. But also even outside of resourcing, I think the lack of leadership in implementing the policies came out very

strongly in our Obesity taskforce Report in 2005 where there was a recommendation that the Department of the Taoiseach, our Prime Minister, should really lead out because of the cross sectoral nature of the solution if you like to obesity, and that didn't happen; it really fell back to the DoH and there hasn't been that cross sectoral approach to tackling obesity and I think that's been replicated in other areas… (Ire 1)

…So you have this scenario of lots of committees and expert bodies and reports and documents and a degree of if you like churning in terms of the policy cycles. But the people with the most expertise and the most commitment having no ability to make decisions and then the people with the ability to make decisions not having the required level of expertise and engagement. (Ire 2)

Yes and food, the food and nutrition lobby is really, really big in Ireland because it is such an integral part of our export. (Ire 3)

…but our challenge really is that industry is very powerful in Ireland and the Irish food industry is even very powerful at European level. Yeah and I mean like we still find, as you're probably aware the other day, the new study citing a causal link between daily consumption of sugary sweets and drinks and obesity, but industry are still kind of saying…well really ignoring it completely and well it's down to individual choice you know? So it's a very frustrating environment. (Ire 4)

Effectiveness of regulatory vs voluntary approaches

Regulatory approaches especially relating to taxing sugary drinks and foods high in saturated fats were perceived as being most effective

No I don't think they are because your day to day with the food sector focuses on increasing sales and profits ultimately and they make token efforts but we have learned this down the years when you're dealing from a public health perspective, whether you're dealing with the tobacco industry or the asbestos sector or the alcohol sector, the food sector is not fundamentally different. They work within whatever regulatory regime is out in place but in the absence of regulation they will maximise return to their shareholders. Regulation is just; the idea of voluntary codes just buys time for the industry. (Ire 2)

Yes absolutely. Public health legislation nearly always has a failed voluntary approach before it, whatever it is - smoking bans were all voluntary - never happened. You bring it in. Drink driving education campaigns, when we brought in eventually random breath testing - completely changed. It was amazing… But where you have legislative issues that are doable as well, then that's clear. (Ire 3)

ITALY

Background

As in many others western countries, Italy is seeing an increase in the proportion of overweight and obese people. A shift away from the traditional Mediterranean Diet has developed, and is reflected by an increase in the prevalence of obesity, especially in the younger population. Obesity rates for females and males are 29% and 18.8% respectively.

Findings

Most Effective Policy Options

Italian key informants varied in their views of the best policy options for their country. Four informants stated that education and information campaigns were needed although not necessarily

the most important option. Three also felt that legislation/regulation and labelling was required and one informant commented upon the current work already being done with industry to reduce salt in bread and pasta and to reduce trans fats:

My opinion we need to do something related to regulation and legislation together for some food goods like for example the trans fats. We do not have any legislation for that so probably we need to do that. And we need to do like we did with smoking habits. And the other thing in my opinion is the labelling of food groups. We didn't have a real policy or law for food labelling so probably we need to change and we need to reformulate our policies for labelling foods. And of course the educational campaigns, but first of all legislation. I think regulation and legislation for some foods and some nutrients like trans fats for example (It 1).

In particular labelling I think is very important. Both implementing a regulation which forces somehow the industry to improve their labelling information on their products and also on the side of the population, making the population more aware…I think the negotiation with the industry for food reformulation in different areas, not just salt, should be implemented and in Italy I am sure there is much to be done in this regard because I know that these negotiations are not as intense and systematic as I think they should be to achieve a good result as soon as possible.(It 2)

I would say that information & education campaigns are still the first, although there are problems but still they have first place because we need to reach the population. Regulation if we have in mind regulation in terms of for instance giving rules for the school meals. In Italy there are no rules for school meals; each school may give different meals and also for instance the presence or not of vending machines in schools is not regulated. So this would be in my opinion important. Labelling is important but after information and education because if you are not informed, you are not able to read the labels.(It 3)

Italy is active in the last year with the food industry to obtain agreement in reformulation of food we are working on salt reduction. We are making agreements with bakers of pasta and the whole industry to reduce the salt in bread. And also we are working with the other producers to reduce salt in other kinds of production. We are working with the food industry also to reduce the content of trans fats in products but we don't have specific agreements to reduce sugar for example in er beverages and soft drinks because we think it is very important for the prevention of obesity and general other problems (It 4).

Most Achievable Policy Options

Reformulation and Education and Information campaigns were perceived as the most achievable policy options, albeit due to actions already being carried out in these areas. Legislation and regulation were also perceived as being achievable if opposition from food companies could be addressed:

…In terms of fat and trans fat, I think … legislation would be achievable because it is quite sensible to discuss that. So we just need some legislation saying that we need to reduce the content of this fat and so on. So probably it should be achieved. Now we don't have any legislation…I mean the problem is always the food companies. They are quite powerful here in Italy so they are probably trying to reach half way between the political will and the will of the food companies. (It 1).

Current Challenges to Implementing Policies

Political will and a lack of resources were identified as the key barriers to developing and implementing policies:

…I think the most important barrier is the political will, as always…so when you say to politicians that it will take 5 years, they say OK but probably I won't be here anymore in 5 years… So the problem first of all is political will. Then of course in this year it is money, lack of money. (It 1)

Well I think that of course there is much to do in this regard. We are only at the very beginning….A working group has been developed to convince the Ministry of Health that we need to develop a full national strategy for reduction of salt intake and this requires the implementation of different activities and different steps. And one of the things which should be done absolutely, but it has not yet been done, is the definition of targets for salt content over different food categories….which are of course very important for negotiation with the food industry, in order that they reformulate their products to reduce the salt content. The problem of the budget is a very important one… we have a widespread economic crisis…and this of course means there has been created a big problem particularly in the field of prevention because when you have to make cuts in the budget you will of course have more difficulty in cutting therapies and treatment which will save lives… (It 4)

Effectiveness of regulatory vs voluntary approaches

All respondents perceived regulatory approaches to be more effective than voluntary ones. Voluntary reformulation was used as an example that had not worked, and regulation on the ban on smoking on public places was given as example of how effective regulation can be. However, comments were made about the difficulty of evaluating the effectiveness of regulation and the need for incentives.

Erm (pause) I think so yes in some of them, related to what we were talking about before for some regulation, we need to do something for health related regulations like smoking campaigns, the smoking ban and the same thing is for trans fats or for fat or for sugar or for salt. We need to do something about the regulation because if we leave it to the voluntary, it will be very, very low. If you say to a person just avoid the trans fats, often times we don't have any effect. (It 2)

In the first instance I would say yes, but this measure, this regulation should be accompanied by for instance subsidies, money. You need to give money to the lower parts of the population to increase for instance the consumption of healthy foods, otherwise I can do what I want in terms of suggested regulations but they will not follow us. But regulation is effective because for instance the example of smoking in Italy. When smoking was banned from cinemas and restaurants and bars and public places, in general it worked. It even worked in Naples which perhaps is not a Nordic city (laughs) but nobody will smoke in restaurants. So regulation is very good and this case however is quite easy to give this regulation - if you smoke you have to pay. But for food, for nutrition it's different. We need incentives to increase the adherence to the rules. (It 4)

MALTA

Background

Although a small island in the central Mediterranean, Malta does not have a diet that is characteristic of the rest of the Mediterranean region. In fact, the Maltese diet has for historical reasons many traits in common with that of Northern Europe. Malta does not have much food production. Most food is imported. MHEC (Ministry for Health, the Elderly and Community Care) has indentified a national "obesity epidemic"; and promotes an inter-ministerial and multi-sectoral approach, so that changes are made to the living environment, which shift it from one that promotes weight gain (obesogenic) to one that promotes healthy choices and a healthy weight for all.

Findings

Most Effective Policy Options
Reformulation, taxation subsidies in combination with education, legislation and regulation was deemed the most effective policy options.

I think erm probably taxation and incentives, so both sides of the...will be effective. There will be elements of effectiveness in that, so I think those will be...I think especially erm things like people are...well they tell us this is all intuitive, not backed by a lot of evidence, but basically the cost of everything is rising and especially fresh fruit & vegetables and so on, have been going up in recent years…(Mal 3)

I'm more inclined to go more for subsidies rather than taxes, at least even as an interim measure. But not subsidies alone. I think there always needs to be education around things, through schools, through the media; the practical hints are so important for people, whatever their age group, practical things to make things easier for them. And obviously part of it was voluntary guidelines for industry and again erm you know I (pause) it's again a complicated thing but ultimately I think regulation in Malta, this is again a bit of a sweeping statement, but when something is legislated I think it kind of has more impact in Malta, rather than sort of voluntary. (Mal 4)

…the reformulation of products will have one of the greatest impacts because if you get reformulation of foods… in Malta we know that people eat a lot of bread so we like get the salt within the bread slowly reduced in the bread they're gonna eat, and that doesn't really need behaviour change because they are eating whatever they are finding available. So we reduce like the trans fats within the products which are available and then they are eating less trans fats. So that maybe one of the easiest approaches but we have to tackle industry. (Mal 5)

Current Challenges to Implementing Policies

A lack of human and financial resources were perceived as the main barrier. Other barriers included a lack of political will, research evidence, collaboration and the power of the food industry.

First it's finance, financing of the action plans that came out of the strategies but another one is also human resources, because being a small country, we have very few resources who work in this area. So that's the next problem. Those are the 2 major problems… (Mal 1)

One of the problems we have at the moment, which is causing us a problem in terms of knowing exactly where to pitch our action and how to prioritise our actions, and actually implement our food and nutrition policy, is that we have no food consumption data. There's no good scientific base of food consumption in Malta. … our first action that needs to be done the moment we get the money next year is to review all our plans and get a proper food consumption survey done and make sure that we have the baseline data on which to pitch our policies and know where to...I mean I can't go to the industry and say we need to cut trans fats if I don't know which industry has the most trans fats and how much trans fats is in the food that they are producing. So we need the data. If we don't have the data...so I think for next year that is going to be our base - getting the data and then building on that. (Mal 2)

Effectiveness of regulatory vs voluntary approaches
Regulatory approaches in the form of taxation and reformulation were perceived as being most effective.

POLAND

Background

Poland is the largest country in central and eastern Europe in both population (38.1 million) and area (312 685 km2). Since the successful transition to a freely elected parliament and a market economy after 1989, Poland is now a stable democracy with constant economic growth and is well represented within political and economic organizations in Europe and worldwide. The transition period of the 1990s saw a considerable improvement in the health status of the Polish population. Average life expectancy at birth reached 80.2 years for women and 71.6 years for men in 2009, but there is still a vast gap in life expectancy between Poland and western EU countries and between life expectancy overall and the expected number of years without illness or disability.

Findings

Most Effective Policy Options

Taxation was perceived as the most effective policy option, with some informants also mentioning reformulation, education and information, labelling, banning trans fats and restricting the marketing of fizzy drinks to children.

…maybe taxation could be most cost effective, however there is a problem of political costs of such activity. Taxing probably products rich in saturated fats. Then I (long pause) I think about products like sweeties which are high in calories and also which are rich in saturated fat and sugar. But I am not quite sure whether it is a good option to tax these products, but maybe. (Pol 2)

Most Achievable Policy Options

A ban on trans fats and salt reduction were perceived as the most achievable and possibly taxing processed foods high in saturated fats.

…I mean a ban on trans fats like in other countries and a reduction of salt, which is very important because hypertension frequency is very high in Poland and consumption of salt is also very high. So they are the two most important and I think realistic targets. Certainly it would be fine to change consumption of sat fats and sugar however I am not sure whether it would be achievable in my country, especially if we think about the tradition of the Polish kitchen, which is rich in sat fats and sugar… (Pol 2)

Current Challenges to Implementing Policies

A lack of political will and financial support were seen as the key barriers to implementing policies.

However sometimes the interest you know of the politicians in Poland is in opposition to the interest, the health interests and in opposition maybe to the EU policy. And for Polish politicians, being oriented on the great number of people living in the country and having their farms and their families, well I mean the government and the parliament members are not eager to change the situation. (Pol 2)

Effectiveness of regulatory vs voluntary approaches

Regulatory approached were seen as being more effective. One informant stated that in order to be effective, political support needs to exist.

Certainly there is the problem of the nanny state and discussion in this area, there are many people who don't accept such regulatory approaches of the government. But from the health point of view, it is a good approach however if supported by the majority of citizens, so first there is a need for political support for such regulatory approaches. And then to do this, that is my opinion. (Pol 2)

PORTUGAL

Background
Research has shown that Portuguese households have reduced their diet quality and decreased their adherence to a Mediterranean food pattern. The first data collection from the Childhood Obesity Surveillance Initiative Portugal, which took place during the 2007/2008 school year across Portugal, with children aged 6–8 years, found prevalence of overweight and obesity to be 32% and 15% respectively based upon the Center for Disease Control and Prevention.

Findings
Most Effective Policy Options
Portuguese informants felt that all policy options were important for improving public health nutrition in Portugal. There was consensus that regulation of food available in schools was a priority, together with legislation regarding fat content in food. Informants also highlighted that there was current activity concerning advertising restrictions, food labelling and food availablity in schools:

I think all the subjects are very important and we are working in almost all of them. Like in advertising, a consensus paper has been written with the food industry about advertising… and in terms of labelling and other issues, it has been until now difficult to legislate so we have worked in terms of consensus, trying to have some consensus. But we will try in the future, when it will be possible to have some legislation… like labelling and the contents of some fat or even salt and so on. We have done that in the schools, in the schools it is not possible to have the fat in food; it is not possible to sell it. And sugar is not possible to sell...that is a regulation for schools. But not for the population in general.(Port 2)

I think legislation regarding maximum theoretical values of fat for instance, those kinds of regulations could be improved in Portugal… Also I think that there is a lot to be done with taxation. If you think of tobacco for instance and although I know this is a very sensitive issue, but I think there is still something that can be done as a result of taxation to regulate a little bit. (Port 3)

Most Achievable Policy Options
The most achievable policy options were identified as being Education and Information campaigns as they were easier to develop and implement. Legislation and food labelling were also deemed important, however education and information campaigns were required to underpin such changes to ensure the population understood and accepted Legislation:

So sometimes you have to have both; not only legislation but also try to have information and education and also try to work with parents and teachers and so on. It's also important to help with labelling because they do not often know how to read the labelling; So we should try to work on labelling but also try to increase literacy on reading labelling... But we are a country where people don't deal very well with rigid regulations. It's one of the problems in the EU in that the cultures are so different and something that is very normal or accepted by some is not so easy for the Portuguese… We have to try and have consensus as well as trying to legislate. But when we have legislation like the tobacco law, we had a lot of people not agreeing and they contest it in the papers and so on but now it is impossible to get it back as it was before the law. (Port 2)

Current Challenges to Implementing Policies
A lack of resources (monetary and human), stakeholder involvement and political will were identified as the key barriers to the development and implementation of policies:

There is a lack of consensus among different stakeholders and it is difficult to motivate the private sector to take action. Political will and the political agenda doesn´t seem to have flexibility on the

implementation of its own policies and this causes a serious problem in implementation and evaluation. Also, the allocation of money is a problem since it is continuously scarce (Port 1)

…I believe that the main barriers will be first the lack of money and resources at this moment and we are in the middle of this crisis and we are very short of money to implement anything new…barriers of money and resources and human resources too. And this is also related to the complex administrative questions at the moment…it is more difficult to implement some projects because bureaucratic and administrative pressures have increased. Other barriers that are more structural barriers,...we don't have a tradition of involving other ministries in these types of programmes. For example the Ministry of Agriculture will be essential to this type of implementation and the national action plan was not used to work with the questions of nutrition and health and they were more interested in the past with funds for farmers and things like that. So ministries like the Ministry of Agriculture and the Ministry of the Environment & Social Affairs, all of them are very important to this but we don't have a tradition of working together involving questions of health and these types of problems. (Port 2)

Probably budget. I think it's safe to say because of the present crisis in Portugal that budget is a huge problem. For you to convince policy makers to invest in such things; it's not the best time to do it so monetary constraints are definitely one problem, one barrier.(Port 3)

Finland, Norway, Denmark and the UK were perceived as having the most effective public health nutrition policies. The named Nordic countries were perceived as having successful polices due to a coordinated approach to development, implementation and monitoring of nutrition policies. The UK in particular was highlighted as having successful salt reduction initiatives by working with industry to reformulate food products:

Finland, Norway and Denmark because these countries have a balance where they include regulation, stakeholder action, proper mechanisms for implementation and for evaluation. Their national policies well describe indicators, targets, timeframe, resource allocation and budget, accountability, and intersectoral action.(Port 1)

I think, well United Kingdom is a good example and in many ways even working with industry, when we are in the meetings or in networks, we know that companies like Kellogg's or others are much more resistant to decrease the salt or sugar in other countries because there is no...the consumers' associations are not so strong in countries like Portugal. So even in some ways, in some issues, we are working together with the Spanish because we have a common culture more or less and some enterprises...but I think UK is a best example in terms of salt as well as the general approach related to nutrition. (Port 2)

Effectiveness of regulatory vs voluntary approaches

All respondents felt that regulatory approaches were more effective than voluntary ones. Reasons included having a greater and automatic impact on the majority of the population and a perception that voluntary actions are not working as the population may know what to do, but choose not to change behaviour.

Well because I think the potential impact is larger, at least in terms of the kind of expenses that the Government would have with regulatory measures but those wouldn't be too big when you compare them to the potential benefits so I think they would be more cost effective I think. (Port 1)

Sometimes people don't comply with voluntary things so it's always progressive changing. It's also what I said to you about the culture. Even people who are ill and they know it's important not to have salt in food, but I always see people in restaurants saying, "I know it's not good, but it's just a little bit." So in terms of culture, it's always possible to have some flexibility and we have to deal with that, so it's not easy…. (Port 3)

SLOVENIA

Background

Findings

Most Effective Policy Options

Legislation and regulation were regarded as the most effective policy options. Informants also commented that legislation and regulation would be most effective with the support of taxation, reformulation, labelling and information and education campaigns.

Legislation and then taxation and erm yeah of course I think everywhere when you can succeed with a systematic approach, when you build a system; you know information is also very important but you know just with information you can't reach much; you have to have the support from the legislation and taxation. I think these two are the most important. (Slov 2)

Legislation would be effective, Well with experience with the tobacco ban, in legislation, let's say the structure of food should be healthy and for example banning the selling of soft drinks in schools would be very easy to achieve. On the other side with taxation of eliminating the use of salt and sugar, so I feel. (Slov 4)

...we already did quite a lot, but for taxation we need the legislation so yes of course yes, there is still room. For example we were also thinking of regulating the advertising the food for children and some restrictions here. (Slov 5)

If you say OK but on my priority list I see that soft drinks are awfully problematic, it's definitely something that I have to legislate on and I have to somehow limit the excess to this product. And on the other side you can probably do something also with taxation. You definitely can go with subsidies because you can't just go pushing down something; you can also increase the important of something else because then you work more on the positive side. If you want to get industry on board then they would be completely resistant if you just legislate; you have to give them a chance to reformulate.(Slov 6)

Most Achievable Policy Options

Banning trans fats and a taxation on products high in salt and sugar in drinks were perceived as being most achievable. Reformulation was also perceived as being achievable, but only with the support of the food industry.

If you want to achieve healthy nutrition concerning consumption of less salt or to consume less trans fatty acids, it's just impossible to achieve if you don't have collaboration of the food industry because sources of salt and trans fatty acids is coming through these unhealthy products. So if they don't collaborate enough you can't achieve this, so legislation could be very supportive, especially when you are tackling some kind of partners who can actually then let's say who are crucial for achieving a healthy offer. (Slov 2)

Well with experience with the tobacco ban, in legislation, let's say the structure of food should be healthy and for example banning the selling of soft drinks in schools would be very easy to achieve.

On the other side with taxation of salt and sugar in drinks and alcohol for example it would be also much easier to achieve eliminating the use of salt and sugar, so I feel. (Slov 4)

Erm (pause) probably the easiest and cost effective would be trans fats really because with quite a limited burden, administrative and all the other burdens, you would get an outcome. (Slov 6)

Current Challenges to Implementing Policies
Political will was seen as the main barrier to implementing policies. Other barriers mentioned were, lack of time and capacity, and the power of the food industry to prevent food polices being developed and implemented.

PLEASE NOTE QUOTES HAVE NOT BEEN PROVIDED AS RESPONDENTS ARE IDENTIFIABLE

Effectiveness of regulatory vs voluntary approaches
Informants felt that either regulatory approaches were more effective, or a combination of both was required.

As usual regulation is more efficient than voluntary because they are forced to do that. Because they have reformulated their products and it costs money and we know that salt is the cheapest erm (pause) preservative. (Slov 4)

Personally I think that regulation is a powerful tool but it depends if it is possible to pass the laws because of the different interests before. Self regulation can be in a way useful and also successful but in some areas it has been proven that it is not, for example in the area of alcohol this doesn't work. So it's very difficult to say. But on the other hand it's very difficult to regulate everything and then it depends how much agreement is amongst the politicians to come with the strong and effective regulations. So it's a trade off all the time. (Slov 5)

COUNTRY	PERCEPTIONS AND VIEWS REGARDING COST EFFECTIVENESS OF REGULATION VERSUS VOLUNTARY MEASURES
**BELGIUM**	SALT REDUCTION AND PREVENTION - COST EFFECTIVE IN THE LONG TERM<br>REGULATION MORE COST EFFECTIVE BUT DIFFICULT TO IMPLEMENT
**CZECH REPUBLIC**	PREVENTION MORE COST EFFECTIVE<br>NUTRITION POLICIES COST EFFECTIVE<br>TAXATION AND REGULATORY APPROACHED MOST COST EFFECTIVE
**ENGLAND**	SATURATED FAT AND SALT MOST COST-EFFECTIVE DIET RELATED RISK FACTORS<br>LABELLING AND LEGISLATION COST EFFECTIVE – UNCERTAIN OF IMPLEMENTATION COSTS<br>REFORMULATION COST EFFECTIVE - LEGISLATION COSTLY
**ESTONIA**	REGULATORY MORE COST EFFECTIVE THAN VOLUNTARY<br>NUTRITION EDUCATION IN SCHOOLS COST EFFECTIVE<br>UNCLEAR WHETHER REGULATORY APPROACH IS MORE COST EFFECTIVE THAN VOLUNTARY<br>NON MEDICAL MORE COST EFFECTIVE<br>REGULATORY APPROACHES MORE COST EFFECTIVE
**FINLAND**	FOCUSING ON HARD FATS (TRANS FAT AND SAT FAT) IS MOST COST EFFECTIVE COMPARED TO OTHER RISK FACTOR<br>CALORIES MOST COST EFFECTIVE<br>COST BENEFIT ANALYSIS OF HEALTH PROMOTION - HEALTH PROMOTION IS COST EFFECTIVE<br>PREVENTION MORE COST EFFECTIVE THAN TREATMENT<br>SCHOOL FRUIT SCHEME NOT COST EFFECTIVE - IEC COSTLY<br>MOST COST EFFECTIVE - HEART GROUPS<br>PROVIDING INFORMATION TO ORDINARY PEOPLE
**GERMANY**	EVIDENCE EXISTS REGARDING TRANS FATS REGULATION REDUCING MORBIDITY/MORTALITY<br>TAXATION MOST COST EFFECTIVE<br>ECONOMIC EVALUATION REQUIRED TO CONFIRM COST EFFECTIVENESS OF LEGISLATION/REGULATION
**GREECE**	VOLUNTARY EASIER TO ADMINISTER THEREFORE MORE COST EFFECTIVE<br>SATURATED FAT, TRANS FAT, SUGAR, SALT AND FRUIT&VEG MORE COST EFFECTIVE THAN CALORIES<br>REGULATION MORE COST EFFECTIVE
**ICELAND**	TAXATION AND REGULATION<br>TRANS FAT BAN<br>LEGISLATION MORE COST EFFECTIVE THAN VOLUNTARY APPROACHES
**IRELAND**	TAXATION, SUBSIDIES, REFORMULATION POTENTIALLY COST EFFECTIVE<br>SALT REDUCTION MOST COST EFFECTIVE FOLLOWED BY TRANS FAT

	REGULATORY APPROACHES MORE COST EFFECTIVE THAN VOLUNTARY APPROACHES
**ITALY**	SALT PERCEIVED AS MOST COST EFFECTIVE DIET RELATED RISK FACTOR<br>TO MEASURE EFFECTIVENESS IN FUTURE IF REGULATION USED<br>UNABLE TO ANSWER WHETHER REGULATION OR VOLUNTARY APPROACHES ARE MORE COST EFFECTIVE
**MALTA**	REGULATION MORE COST EFFECTIVE THAN VOLUNTARY ACTIONS<br>SALT REFORMULATION - COST EFFECTIVE
**POLAND**	TARGETING FRUIT AND VEG IS COST EFFECTIVE, GOOD FOR THE INDUSTRY AND GOOD FOR THE PEOPLE<br>TAXATION COULD BE MOST COST EFEFCTIVE<br>REGULATORY APPROACHES MORE COST EFFECTIVE THAN VOLUNTARY APPROACHES ESPECIALLY WITH POLITICAL SUPPORT
**PORTUGAL**	TAXATION AND REGULATION ARE MOST COST EFFECTIVE<br>REGULATORY APPROACHES COST EFFECTIVE FOR REDUCING SALT AND SAT FAT
**SLOVENIA**	MANY SUBSIDIES NOT COST EFFECTIVE - SCHOOL MILK SCHEME NOT COST EFFECTIVE - TOO BUREAUCRATIC;<br>LEGISLATION COST EFFECTIVE, SOFT APPROACHES SUCH AS LABELLING AND IEC NOT COST EFFECTIVE<br>PREVENTION MORE COST EFFECTIVE<br>NATIONAL PLAN FOCUSED ON PREVENTION - COST EFFECTIVE<br>REGULATORY APPROACHES MORE COST EFFECTIVE THAN VOLUNTARY APPROACHES<br>TAXING SUGARY DRINKS/PRODUCTS COST EFFECTIVE<br>IEC NOT COST EFFECTIVE,<br>LEGISLATION/ REGULATION MOST COST EFFECTIVE, IEC QUITE EXPENSIVE, LEGISLATION AND REGULATION COST EFFECTIVE, TRANS FAT COST EFFECTIVE, TAXATION COULD HAVE LARGE ADMINISTRATIVE BURDEN SO NOT COST EFFECTIVE IN THIS WAY

COUNTRY	PERCEPTIONS AND VIEWS REGARDING WHAT NEEDS TO BE DONE TO IMPROVE PUBLIC HEALTH NUTRITION IN THE 14 COUNTRIES
**BELGIUM**	REGULATE SAT FAT AND SUGAR CONTENT<br>USE LEGISLATION TO GET REFORMULATION<br>SUBSIDIES FOR HEALTHY FOOD/ SCHOOL MEALS<br>REFORMULATION IS A PRIORITY<br>KEEP SALT LEGISLATION<br>DETAXATION OF HEALTHY FOOD<br>LEGISLATION FOR EDUCATIONAL PROGRAMMES IN SCHOOLS, SUBSIDIES/DETAXATION<br>ONGOING TARGETED INFORMATION AND EDUCATION CAMPAIGNS
**CZECH REPUBLIC**	LEGISLATION ON NUTRITIONAL RECOMMENDATIONS FOR SCHOOL CANTEENS<br>BAN VENDING MACHINES<br>LABELLING

	TAXATION FOR JUNK FOOD<br>EDUCATION IN SCHOOLS
**ENGLAND**	COMMON AGRICULTURAL POLICY REFORM, PRE-SCHOOL NUTRITION AND TAXATION<br>REGULATION, LEGISLATION AND REFORMULATION AS IT HITS THE ENTIRE POPULATION RATHER THAN INDIVIDUALS<br>TRAFIC LIGHT LABELLING - MAKING INFORMATION UNDERSTANDABLE<br>POSSIBLY SUBSIDIES<br>BAN ADVERTISING OF JUNK FOOD AL TOGETHER<br>INCREASE TAXATION ON UNHEALTHY FOODS<br>AGRICULTURAL SUBSIDIES TO PROMOTE FRUIT & VEG<br>LEGISLATION FOR REFORMULATION AROUND SALT AND TRANS FAT<br>REDUCE SAT FAT BY USING CAMPAIGNS, LABELLING AND POSSIBLY LEGISLATION
**ESTONIA**	LEGISLATION, REGULATION REGARDING CHILDRENS NUTRITION, LABELLING AND IEC<br>SUBSIDIES, REFORMULATION, LEGISLATION FOR SCHOOLS<br>LEGISLATION, SUBSIDIES AND IEC (NUTRITION CURRICULUM)<br>LEGISLATION, REGULATION AND SUBSIDIES
**FINLAND**	REFORMULATION, LEGISLATION AND REGULATION, LABELLING AND IEC<br>TAXATION OF UNHEALTHY PRODUCTS<br>SUBSIDISE FRUIT & VEG<br>GUIDING CATERING IN PRE-SCHOOLS, SCHOOLS AND WORK PLACES COMBINED WITH EDUCATION<br>MORE REGULATION WITH SALT<br>TAXATION FOR SAT FAT, SALT AND SUGAR
**GERMANY**	TRAFIC LIGHT LABELLING (BLOCKED AT THE MOMENT)<br>ALL OPTIONS POSITIVE HOWEVER TAXATION IS PERCEIVED AS MOST DIFFICULT<br>LEGISLATION, REGULATION, SUBSIDIES, LABELLING, REFORMULATION AND IEC
**GREECE**	TAXATION<br>REFORMULATION<br>LABELLING<br>LEGISLATION/REGULATION REGARDING ADVERTISING OR FOOD TO CHILDREN<br>REGULATION FOR FOOD ALLOWED IN SCHOOLS<br>REDUCE TAXATION ON FRUIT &VEG AND INCREASE ON UNHEALTHY SNACKS<br>IEC
**ICELAND**	LEGISLATION AND REGULATION ON TRANS FAT<br>TAXATION AND REGULATION

	LABELLING<br>IEC
**IRELAND**	LEGISLATION AROUND FOOD MARKETING TO CHILDREN<br>IMPROVED FOOD LABELLING - TRAFIC LIGHT SYSTEM<br>SUGAR TAX<br>TAXATION OF SUGARY DRINKS<br>LEGISLATION, TAXATION (ON SUGAR OR FATS) AND SUBSIDIES
**ITALY**	REFORMULATION<br>LEGISLATION/REGULATION - ESPECIALLY TRANSFATS<br>REFORMULATION - ALREADY WRKING WITH INDUSTRY TO REDUCE SALT IN BREAD/PASTA AND REDUCE TRANS FATS<br>REGULATION (SCHOOL MEALS)<br>LABELLING<br>IEC
**MALTA**	IEC CONSTANTLY TO REMIND THE PUBLIC
**POLAND**	TAXATION (E.G. SAT FAT, SUGAR)<br>LABELLING<br>TRANS FAT BAN<br>RESTRICTIONS IN MARKETING OF FIZZY DRINKS TO CHILDREN
**PORTUGAL**	REGULATION OF FOOD AVAILABLE IN SCHOOLS<br>LEGISLATION REGARDING FAT CONTENT<br>TAXATION SIMILAR TO TOBACCO<br>REFORMULATION AND IEC
**SLOVENIA**	LEGISLATION FOR SALT AND TRANS FAT REFORMULATION<br>REGULATE FOOD ADVERTISEMENTS TO CHILDREN<br>SUGAR EITHER TAXATION OR LEGISLATION<br>SUBSIDIES<br>GIVE FOOD INDUSTRY A CHANCE TO REFORMULATE VOLUNTARILY<br>LABELLING
